# Background splicing as a predictor of aberrant splicing in genetic disease

**DOI:** 10.1080/15476286.2021.2024031

**Published:** 2022-02-19

**Authors:** Alexieva D, Long Y, Sarkar R, Dhayan H, Bruet E, Winston Rm, Vorechovsky I, Castellano L, Dibb N.J

**Affiliations:** a Department of Metabolism, Digestion and Reproduction, Institute of Reproductive and Developmental Biology, Imperial College London, London, UK; b Faculty of Medicine, University of Southampton, Southampton, UK; c Department of Surgery and Cancer, Imperial College London, Imperial Centre for Translational and Experimental Medicine (Ictem), London, UK; d School of Life Sciences, University of Sussex, Falmer, UK

**Keywords:** Cryptic splice site, exon skipping, background splicing, pseudoexons, recursive splicing, cancer

## Abstract

Mutations of splice sites, auxiliary splicing elements and the splicing machinery cause a wide range of genetic disease. Here we report that many of the complex effects of splicing mutations can be predicted from background splicing information, with emphasis on BRCA1, BRCA2 and DMD. Background splicing arises from very low level splicing between rarely used background splice sites and from low-level exon skipping between intron splice sites. We show how this information can be downloaded from the Snaptron database of spliced RNA, which we then compared with databases of human splice site mutations. We report that inactivating mutations of intron splice sites typically caused the non-mutated partner splice site to splice to a known background splice site in over 90% of cases and to the strongest background splice site in the large majority of cases. Consequently, background splicing information can usefully predict the effects of splice site mutations, which include cryptic splice activation and single or multiple exon skipping. In addition, de novo splice sites and splice sites involved in pseudoexon formation, recursive splicing and aberrant splicing in cancer show a 90% match to background splice sites, so establishing that the enhancement of background splicing causes a wide range of splicing aberrations. We also discuss how background splicing information can identify cryptic splice sites that might be usefully targeted by antisense oligonucleotides (ASOs) and how it might indicate possible multiple exon skipping side effects of ASOs designed to induce single exon skipping.

## Introduction

Human genetic disease is frequently caused by mutations that disrupt intron splice sites, auxiliarly splicing motifs or the splicing machinery [1], it is estimated that 50% of all deleterious mutations cause aberrant splicing for large genes with many exons [2]. It is important but still challenging for *in silico* programmes to identify variants in patients that disrupt splicing and to predict the effect of such splicing mutations [2–5].

**Figure 1.**
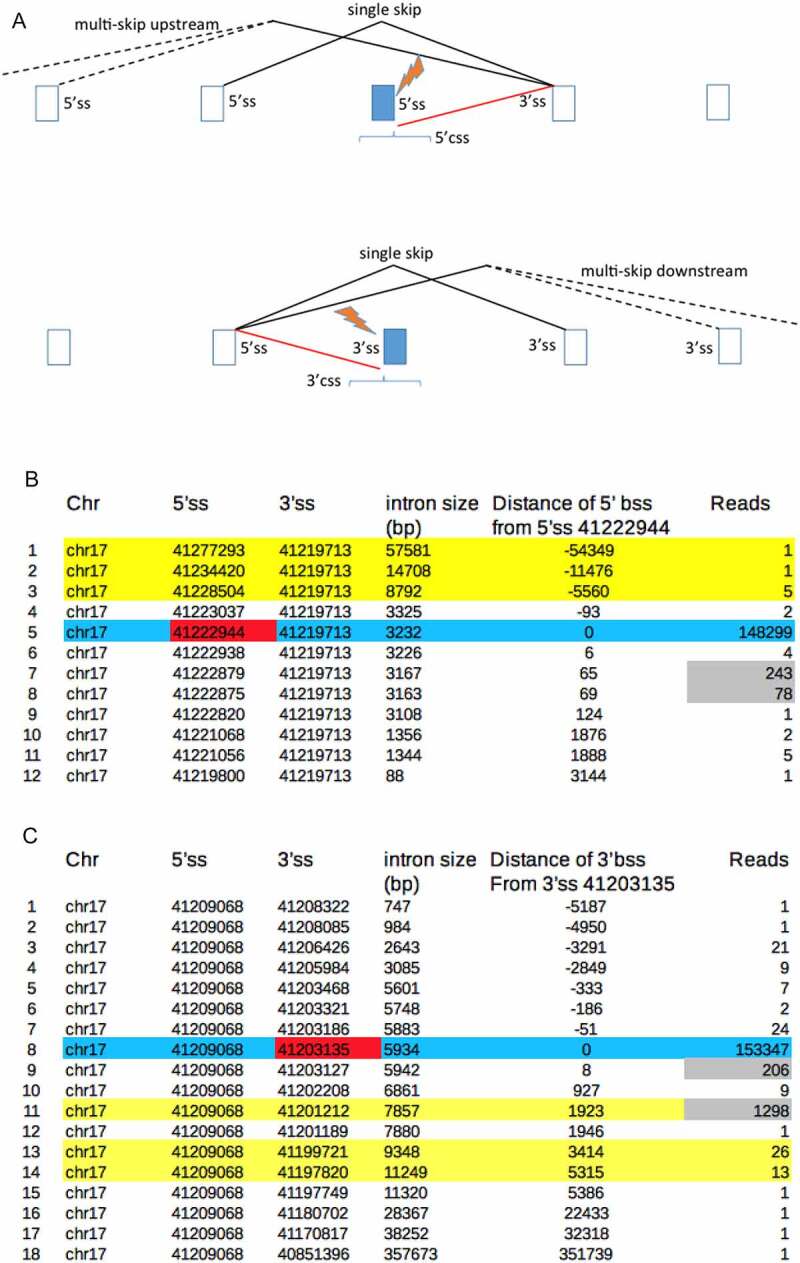
**A**. Aberrant splicing events that are commonly activated by mutations of the 5ʹ or 3’ss of introns. The brackets reflect that most activated css lie within 1000 bases of the ss mutation. **B,C**. Splice site mutations activate background ss (bss), particularly those with the most reads. **B**. The Snaptron data is arranged to show all splicing events involving the 3’ss 41,219,713 of wild type BRCA1 (see text). Blue shading shows normal splicing, yellow shading shows background exon skipping, red shading shows the mutated 5’ss partner 41,222,944 and grey shading shows the reads of the bss prior to their further activation by this mutation. **C**. All splicing events involving the 5’ss 41,209,068, blue and yellow shading as above, red and grey shading indicates the effect of mutation of the normal partner 3’ss 41,203,135.

Mutations of intron splice sites often cause the vigorous activation of nearby dormant cryptic splice sites, which are used instead of the mutated intron splice site [6](Figure 1A). We previously established that css are already active, albeit at very low levels, in normal genes. We did this by using expressed sequence tags (ESTs) to identify rare splice sites and then compared their positions to known css that are activated in human disease [7]. However, this approach was limited to a minority of genes for which there was sufficient EST data. Since that time a large amount of RNA-sequencing data has been deposited, which we reasoned would strongly increase the power of css prediction. In support of this, RNA sequencing studies have shown that splicing is accompanied by a background of low level or noisy splicing at a large number of hidden splice sites within introns and exons [8].

The Snaptron database lists all of the RNA-seq reads from over 70,000 human samples that were most probably generated by splicing [9]. As expected [8], there are far more splicing events with very low reads (background splicing) in the Snaptron database than there are splicing events with the high reads required for intron removal and functional alternative splicing (Figure 1B, C, Appendix 1). The Snaptron database shows that background splicing occurs between: 5ʹ and 3ʹ background splice sites located throughout exons and introns; from low-level exon skipping between intron splice sites and from low-level splicing between bss and intron ss. Here we compare the Snaptron database to cryptic splice site and exon skipping databases [6,10] and conclude that background splicing determines the effect of splicing mutations upon exon skipping as well as css activation. Further comparisons show that bss in normal human genes are also used for de novo splice site and pseudoexon formation, recursive splicing and aberrant splicing in cancer. We also discuss how background splicing information can inform splicing therapy.

## Materials and methods

Experimental reports of mutations that cause aberrant splicing of BRCA1, BRCA2 and DMD were obtained from the database of aberrant splice sites (DBASS) the human genome mutation database (HGMD), the Leiden Open Variation Database online (LOVD) and by searching PubMed [6,11,12].

We used the BLAT tool [13] from UCSC website http://genome.ucsc.edu/ [14] to obtain genome reference numbers for relevant splice sites.

We then compared the above experimental databases of aberrant splicing to the Snaptron database of spliced RNA sequences [9]. We downloaded Snaptron data for individual genes in a manner that allowed us to identify background splicing events that might be activated by splicing mutations (Figure 1(A) and Appendix 1).

Snaptron has four different RNA sequencing databases that can be analysed. SRAv1 (hg19) and SRAv2 (hg38) are from the sequencing read archive at NCBI and contain 41 and 83 M splice junctions identified by sequencing, respectively. There are also two smaller databases TCGA (hg38) and GTEx (hg38) with 37 and 29 M junctions [9].

Statistical analysis. Probability values for Table 2 were obtained by binomial distribution analysis (see Table S2 css) and for Table 3 by a Pearson chi-square test for the 5’ss data (rows 1, 5) and Fisher’s exact t-test for the 3ʹ ss data (rows 3, 7).

**Table 1.** Comparison of the experimental effect of splice site mutations of BRCA1 with snaptron splicing data. Column A lists the mutated intron 5ʹ or 3ʹ splice sites (chr17hg19). Column B shows whether the mutation caused css activation, exon skipping or both, the position of the css relative to the mutated intron ss is also indicated. Columns C-G compare RNA sequencing data from Snaptron. Column C indicates whether the experimentally identified css (from column B) exactly matches a background splice site in Snaptron. Column D shows the css rank, for example 1(4) for row 3 of this column means that Snaptron identified four background splice sites within 1000 bp (upstream or downstream) of the 5’ss 41,258,472 and that the site that matched the experimentally identified css had the most reads. Column E lists the reads for the highest scoring bss within 1000 bases of the mutated splice site. For rows 5, 24, 31 and 35 the reads for the bss that matches the css is given in brackets. Columns F and G list background reads for single and double exon skipping. The shaded boxes indicate the RNA splicing reads that are not a good fit to the experimental data, these are discussed in Table S1, which also lists references

	A	B	C	D	E	F	G
	Mutated splice site	Experimental	css	snaptron	snaptron	snaptron	snaptron
	5ʹ ss	summary	match in	css rank	bss reads	single skip	double skip
			snaptron			reads	reads
1	41,276,033 (exon 2)	skip			11	898	0
2	41,267,742 (exon 3)	skip			24	2622	104
3	41,258,472 (exon 5)	Css(−22) & skip	Yes	1(4)	7375	3629	178
4	41,256,884 (exon 6)	Css (−9)	Yes	1(5)	327	5	6
5	41,256,138 (exon 7)	Css (−62)	No	0(1)	(0)1	3	2
6	41,251,791 (exon 8)	skip			0	83	0
7	41,249,260 (exon 9)	skip			31	2193	3
8	41,243,451 (exon 11)	skip (and alt ss enhancement)			2	346	44
9	41,242,960 (exon 12)	skip			115	1	18
10	41,234,420 (exon 13)	skip			0	164	0
11	41,228,504 (exon 14)	single and double skip (weak)			1	971	58
12	41,226,347 (exon 15)	Css (−11), single and double skip	Yes	1(1)	494	476	356
13	41,222,944 (exon 16)	Css (65,69)	Yes, yes	1(5), 2(5)	243, 78	0	5
14	41,219,624 (exon 17)	Css (153, weak) & skip	Yes	1(3)	36	1030	1
15	41,215,890 (exon 18)	skip			179	140	17
16	41,215,349 (exon 19)	skip			23	1	56
17	41,209,068 (exon 20)	Css (87) & skip	Yes	1(1)	19	52	0
18	41,203,079 (exon 21)	skip			4	1298	0
19	41,201,137 (exon 22)	Css (156,weak) & skip	Yes	1(1)	6	2732	26
20	41,199,659 (exon 23)	Css (5, weak) & skip	Yes	1(2)	247	300	132
	3’ss						
21	41,267,797 (exon 3)	Css (7)	Yes	1(3)	5	2622	178
22	41,258,551 (exon 5)	skip			18	3629	6
23	41,256,974 (exon 6)	Css (−59)	Yes	1(4)	15	5	2
24	41,256,279 (exon 7)	Css (−10)	No	0(1)	(0)81	3	0
25	41,251,898 (exon 8)	Css (−69)	Yes	1(1)	4	83	3
26	41,247,940 (exon 10)	skip			339	840	44
27	41,246,878 (exon 11)	skip & alt skip			3	206,364	18
28	41,219,713 (exon 17)	skip			12	1030	17
29	41,215,969 (exon 18)	skip			11	140	56
30	41,215,391 (exon 19)	skip			1	1	0
31	41,209,153 (exon 20)	Css (13,weak) & strong skip	Yes	2(2)	(1)3	52	0
32	41,203,135 (exon 21)	Css (8,weak) & skip	Yes	1(5)	206	1298	26
33	41,201,212 (exon 22)	skip			1147	2732	132
34	41,199,721 (exon 23)	skip			26	300	0
35	41,197,820 (exon 24)	Css (11)	Yes	2(4)	(5)26	0	0

**Table 2.** Summary of matches between 5ʹ and 3ʹ css from DBASS, BRCA1, BRCA2 and DMD (columns 1,2) with background splice sites from Snaptron (columns 3 to 5). Data summarized from the Tables listed in source column 6

**5ʹcss**	No. of css	No that match	Top match	Poor match	Source
	analysed	snaptron bss			
DBASS5	237	201 (85%)	150 (75%)	9	Table S2
DBASS5w	14	11	10	2	Table S2
BRCA1	10	9	8	1	Table 1
BRCA2	5	4	2	1	Table S2
DMD	13	4	1	5	Table S2
**3ʹcss**					
DBASS3	110	97 (88%)	62 (64%)	2	Table S2
DBASS3w	39	38	31	0	Table S2
BRCA1	7	6	4	1	Table 1
BRCA2	13	10	6	2	Table S2
DMD	9	6	2	1	Table S2

**Table 3.** Css activation versus exon skipping. The experimental results listed in column A are summarized from the cryptic splice site database DBASS (Table S2) and an exon skip database (Table S3) and they show the numbers of reports of css activation only, exon skipping only or both in response to 5ʹ or 3’ss mutations. Columns B and C are from Snaptron and show how the samples divide with respect to the relative number of reads for single exon skipping versus the number of reads for the bss that matches the css. For examples that do not report a css or more rarely report a css that does not match a bss we used the read numbers of the top bss (bss with the most reads within 1000 bp of the mutated ss). Columns D and E show the total css and single exon skip read count (Tables S2, S3). Shaded examples are discussed (Tables S2, S3, see text)

	A	B	C	D	E
	Experimental results	Snaptron data			
		skip>css	css>skip	Total css	Total skip
		reads	reads	reads	reads
	**DBASS5 (Table S2)**				
1	70 css only	11	59	105,757	6884
2	36 css + skip	24	12	10,112	143,955
	**DBASS3 (Table S2)**				
3	18 css only	2	16	26,786	2659
4	22 css + skip	11	11	15,839	31,527
	**5ʹ skip database (Table S3)**				
5	79 skip only	71	8	5978	217,587
6	3 skip + css	1	2	9395	2852
	**3ʹ skip database (Table S3)**				
7	64 skip only	54	10	17,346	349,439
8	4 skip + css	2	2	1939	3185

## Results

### BRCA1

We initially analysed BRCA1 as proof-of-principle because its mutational landscape in cancer is well described and includes splicing mutations that have been repeatedly analysed [15,16]. Figure 1A illustrates that mutations of intron splice sites typically activate css or exon skipping. Figure 1B,C show that the aberrant splicing pathways illustrated in Figure 1A already occur at low background levels in normal BRCA1.

Figure 1B lists all of the 5’ss partners for the 3’ss 41,219,713 (hg19) of intron 16 of BRCA1 and their read numbers, as listed in Snaptron. As expected there are a large number of reads (148,299) for splicing between 3’ss 41,219,713 and its normal 5’ss partner 41,222,944 of BRCA1 (blue shading). Other 5’ss partners of the 3’ss 41,219,713 are also used but at much lower background levels. These include single and multiple exon skipping events (yellow shading) between the 3’ss 41,219,713 and the 5’ss of other upstream introns. In addition there are 2 reads for a rare splicing event between 3’ss 41,219,713 and an exonic 5’ss that is located −93 bases upstream of the normal 5’ss 41,222,944 and further low level reads for seven background 5’ss that are located downstream within the intron.

Mutation of BRCA1 5’ss 41,222,944 of intron 16 is reported to activate a css at either +65 [17–19] or at +69 [20] These two css exactly match the bss of normal BRCA1 with the most supporting reads (Figure 1B, grey shading). The background splicing information is therefore a very good match to and helps to explain why different css were identified between groups.

Similarly, Figure 1C compares the known effect of mutation of the 3’ss 41,203,135 (hg19) of intron 20 of BRCA1 (red shading) with the background splicing events involving its normal partner 5’ss 41,209,068, as illustrated in Figure 1A (bottom diagram). Mutation of the 3’ss 41,203,135 is known to activate single exon skipping between the normal partner 5’ss 41,209,068 and the downstream intronic 3’ss 41,201,212 plus weaker activation of a 3ʹcss 41,203,127 at +8 [17,18]. Figure 1C shows that these two aberrant splicing events also have the most background splicing reads in normal BRCA1.

The key data from Figure 1B and C is summarized in Table 1 (rows 13 and 32), which compares the reported effects of all mutations of the intron splice sites of BRCA1 with Snaptron data. Figure S1 shows this data in full in the same format as Figure 1B, C. From the literature we identified 17 different css that are activated by mutations of the indicated BRCA1 splice sites and Table 1 column C shows that 15 of these css exactly match bss of wild-type BRCA1, the two exceptions are shaded in column C and discussed in Table S1. Twelve of the 15 bss that match css have the highest reads of all candidate css, as listed under column D and as illustrated in Figure 1B, C.

Seventeen out of 35 of the splice site mutations of BRCA1 in Table 1 activate exon skipping rather than css and eight of the splice site mutations do both (Table 1, column B). The ratio of css reads to exon skip reads from the background RNA splicing data (Table 1, columns E, F) appears to correlate with the experimental finding of whether splice site mutations activate css or exon skipping. There are seven exceptions to this that are shaded as pairs in columns E & F and are discussed (Table S1). Also shaded are some possible false-positive bss reads for both css activation (column E rows 5, 24, 31 and 35) and for a double exon skip (column G row 16), see Table S1 and Discussion. Table 1 indicates that the effect of splice site mutations upon css activation and even exon skipping can be inferred from background splicing data. In order to test this hypothesis we analysed a further 300 medical syndromes caused by splice site mutations.

## Further css analysis

We next compared the Snaptron database with the database of aberrant splice sites (DBASS). DBASS lists the experimental results for splicing mutations that cause a wide range of human genetic diseases [6]. We first compared the DBASS5 experimental results for 5ʹ css activation with the Snaptron RNA splicing data. Table S2 (Index) shows how 199 of the 459 mutations in DBASS5 that activate 5ʹcss were systematically chosen to cover every listed medical syndrome. We generated similar tables of background splicing to those illustrated in Figure 1 for each of the 199 mutations and compared these with the experimental results. The analysis of each mutation is summarized in single rows in Table S2 (css). Table 2 row DBASS5 summarizes Table S2 (5ʹcss) and shows that 201 out of 237 of the 5ʹcss identified by experiment (some mutations activate more than one css) exactly match bss in Snaptron and are therefore already in use at low levels by normal genes. Table 2 column 4 shows that 150 of the 237 reported css matched bss that have the greatest number of supporting reads compared to other bss (p = 1 × 10^−56^). Similar results were found for the analysis of the 3ʹcss listed in DBASS3 where 97 out of 110 3ʹcss matched bss in Snaptron and 62 matched bss with the highest reads (p = 3.2 × 10^−23^).

The reason why 15% or so of the experimentally identified 5ʹ css or 3ʹcss did not match a bss was usually because there were no bss reads for comparison (Table S2). Where bss data were available, we found that bss did not match the experimentally reported 5ʹ or 3ʹ css in only 2 to 3% of cases, listed as poor matches in Tables 2 and S2 (css). Table 2 also includes summaries for similar analyses of BRCA1 (Table 1), BRCA2 and DMD (Table S2). DBASS5w and DBASS3w of Table 2 summarize an analysis of a subcategory of css from DBASS that are activated by relatively weak mutations that occur outside the most conserved regions of the normal 5ʹ or 3’ss (Table S2). The activated css of DBASS5w and DBASS3w tend to match bss with particularly high reads (Table S2). Overall at least 85% of css originate from bss and usually css match bss with the most reads relative to other bss candidates (Table 2).

## Exon skipping

We next asked whether background splicing data can indicate whether splice site mutations might cause exon skipping rather than css activation. Some of the papers referenced in DBASS report clearly whether or not exon skipping accompanied css activation (Table S2 css column N). Table 3 column A rows 1 and 2 summarize that there are 70 reports of css activation only and 36 reports of both exon skipping and css activation for the 5’ss mutations analysed in Table S3. For the reports of css activation only, the total number of background single exon skip reads from the 70 examples is 6884, which is much smaller than the total background skip reads (143,955) from the 36 reports of both css and skip activation, so confirming the correlation seen for Table 1. Similar results were found for DBASS3 (Table 3, rows 3 and 4).

Table 3 also summarizes an analysis of a second database of splicing mutations (Table S3) that generally cause exon skipping rather than css activation [10]. Table 3 row 5 shows that we analysed 79 experimental reports of 5’ss mutations that cause exon skipping only. Of these, 71 examples have higher background splicing reads for exon skipping than reads for potential css. Conversely, the experimental reports in DBASS5 of 5’ss mutations that only caused css activation (column A, row 1) had higher reads for the css than for background exon skipping in 59 out of 70 examples (6 × 10^−19^). Table 3 shows that similar results are found by comparing the 64 examples of 3’ss mutations that cause exon skipping only (row 7) with the 18 examples of 3’ss mutations in DBASS3 (row 3) that cause css activation only (p = 1.4 × 10^−8^). Overall these results confirm that the likely effect of splicing mutations upon css activation or exon skipping can in general be inferred from their background splicing ratios. The exceptions to this general finding are shaded in Table 3 and discussed in more detail in Tables S2 and S3. This analysis shows that when the background reads for single exon skipping are greater than the background reads for any candidate css, then exon skipping usually occurs in response to a splice site mutation (Figure 1(A)).

## Multiple exon skipping

Table 4 lists all experimental reports of multiple exon skipping events that we found and compares these to the background splicing reads from Snaptron. We also included experiments that did not detect the multiple skipping events indicated by Snaptron but used RT-PCR primers that were capable of doing so (rows 33 to 42). We did not include predictions of multiple exon skipping from Snaptron where experiments were restricted to single skip analyses.

**Table 4.** **Multi-exon skipping events**. Experimental reports of mutations that cause multi-exon skipping compared to background splicing predictions. Genes are listed in column B and the experimental results are listed in column C and also column F. Snaptron data is compared in columns D, E and G to I. For shading see text

A	B	C	D	E	F	G	H	I
Splice site mutations that cause multiple exon skipping			Snaptron data					
	Gene	Experimental effect	Single skipreads	Double skipreads	css	css match	css reads	Source
1	LAMP2A	Single and double exon skip (similar ratio).	64	80	no			Appendix 1
2	LAMP2B	weak css and strong single exon skip, no double skip	8	0	yes	×	0	Appendix 1
3	LAMP2C	weak single exon skip and strong double exon skip	1	25	no			Appendix 1
4	p67-PHOX	css, single and double skip, relative ratios not given	26	1	yes	√	3	Table S2 5ʹcss
5	PKLR	css, single (major event) and double skips	0	0	yes	×	0	Table S2 5ʹcss
6	ATP7A	css, single (major event) and double skips	340	84	yes	√	24	Table S2 5ʹcss
7	COL5A1	css (x2, weakest), exon skip, double exon skip (major)	1	60	yes	√√	10,6	Table S2 3ʹcss
8	HPRT1	css (20%), exon skip (60%), double skip (20%)	26	914	yes	√	410	Table S2 3ʹcss
9	ALDH3A2	Single and double exon skip (strongest)	748	4364	no			TableS3 5’skip
10	ATM	Single skip (90%) and double skip (10%)	734	56	no			TableS3 5’skip
11	CAPN3	Double exon skip reported (single skip unclear)	8	2	no			TableS3 5’skip
12	ECHA	Single (major) and double exon skip (minor)	22	285	no			TableS3 5’skip
13	NTRK1	Single (stronger) and double exon skip.	9	42	no			TableS3 5’skip
14	SEDL	single and double exon skip (ratio not clear).	194	135	no			TableS3 5’skip
15	WT1	Single and double exon skip (similar amounts).	0	6	no			TableS3 5’skip
16	ALDH3A2	Single and double exon skip. Ratio not given	6111	1536	no			TableS3 3’skip
17	ATM	Single and double exon skip. Ratio not given.	1052	683	no			TableS3 3’skip
18	BTK	Triple exon skip and css only	1	11, 27 (triple)	yes	√	66	TableS3 3’skip
19	KCNQ1	Double exon skip only.	0	295	no			TableS3 3’skip
20	BRCA1	Single and double skip (weak)	971	58	no			Table 1
21	BRCA1	css, single (major events) and double skip (minor)	476	356	yes	√	494	Table 1
22	BRCA2	Single and double skip (major effect for 1 of 2 reports)	4	62	no			Table S4
23	BRCA2	Single and quadruple skip	56	22, 144, 194(quad)	no			Table S4
24	BRCA2	Single and double skip (major effect)	14	64	no			Table S4
25	BRCA2	Single and double skip (minor)	37	8	no			Table S4
26	DMD	double skip only	7	83	no			Table S5
27	DMD	skip and double skip (ratio not given)	4	67	no			Table S5
28	DMD	skip and double skip (ratio not given)	19	0	no			Table S5
29	DMD	css, skip (strongest) and double skip (weakest)	92	1	yes	√	12	Table S5
30	DMD	skip, double skip, triple skip (ratio not clear)	0	21, 6 (triple)	no			Table S5
31	DMD	css, skip, double skip (ratio not clear)	13	1	yes	√	1	Table S5
32	SLC35A1	css (major) single skip, double skip (weakest)	2919	650	yes	√	26,028	Table S6 5ʹcss
33	FGA	multiple css reported but no single or double exon skipping	1	11	yes	×	1,0,3	Table S3 5ʹcss
34	COL5A1	Single and double exon skips not reported	10	27	yes	×	0	Table S3 5ʹcss
35	STK11	only css reported	0	2058	yes	√	79,56	Table S3 3ʹcss
36	COL7A1	only a css reported	895	494	yes	×	151	Table S3 3ʹcss
37	FBN1	Single exon skip only	9	55	no			Table S3 3’ss
38	BRCA1	css at −62 reported but not single or double exon skipping	3	2	yes	√	0	Table 1
39	BRCA2	css and a single exon skip reported but not a double skip	15	390	yes	√	0,23	Table S4
40	DMD	single exon skip reported	0	8	no			Table S5
41	DMD	single exon skip reported but not a triple skip	3	0, 10(triple)	no			Table S5
42	DMD	css and single exon skip but not a double skip reported	11	21	yes	√	1	Table S5

The first three examples of Table 4 are taken from a report about proteins LAMP2A, B and C which are generated by alternative splicing from a common 5’ss and three alternative 3’ss [21]. The authors report that the same mutation of the common 5’ss has different effects upon single or double exon skipping by each 3ʹ alternative ss. It can be seen that these differences in skipping correlate well with the relevant background splicing reads (Table 4, Appendix 1). Other notable features of Table 4 include reports of double exon skips only (rows 19 and 26) or mainly double exon skipping (rows 3, 7, 9, 22 and 24) and how this correlates with the higher background reads for double skips than single exon skips in Snaptron. Similarly the reports of css and triple exon skipping (row 18) and single and quadruple exon skipping (row 23) are a good match to the background splicing reads.

There are ten examples (rows 33 to 42) in Table 4 where the experimental results do not match the multiple exon skip predictions from Snaptron and seven examples (8, 12, 13, 15, 18, 28 and 30) where there is some but not exact agreement. There are also six css listed that did not match bss. For the css of row 5, Snaptron has no bss with which to compare and for row 2 the css has a non-consensus sequence, which is filtered from Snaptron [9]. The other four non-matching css are discussed at the bottom of the source tables. This analysis shows that high background reads for multiple exon skips is a reasonable indication that these events will occur in response to splice site mutations.

## Other aspects of splicing

**Figure 2.**
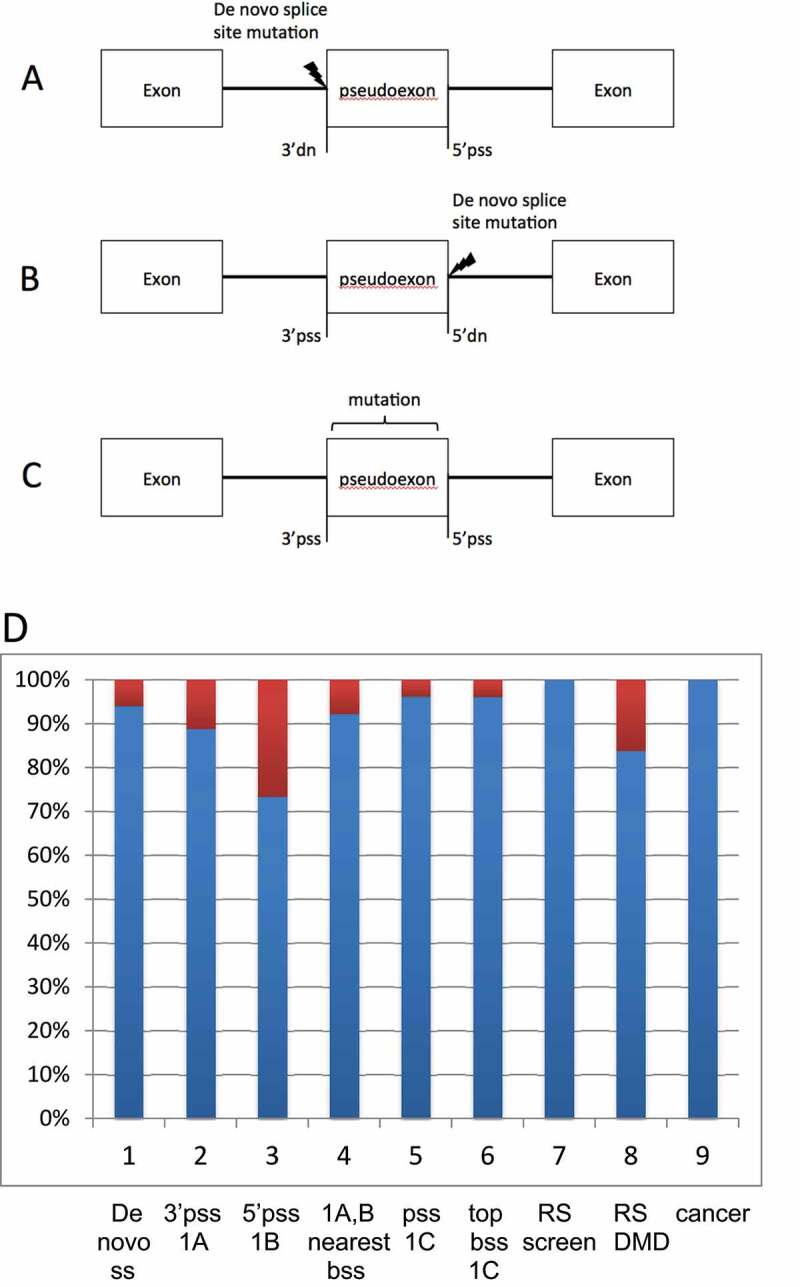
A-C. The three most common ways of generating a pseudoexon [9]. A) A 3ʹ de novo mutation activates a downstream 5ʹ pseudo splice site. B) A 5ʹ de novo mutation activates an upstream 3ʹ pseudo splice C) Mutations other than de novo splice site mutations can enhance pseudoexon usage, of these the most common mutations occur within the pseudoexon. D) Match between background splices sites (bss) with de novo splice sites, pseudoexon ss (pss), recursive ss (RS) and aberrant ss in cancer (Tables S4, S5). Columns **1**: 47/50 match between bss and ‘enhanced’ de novo ss. **2, 3**: 63/71 and 14/22 match between bss and the 3ʹ or 5ʹ pss of pseudoexons type I (Figure 2(A,B)). **4**: 71/77 bss that match the pseudo ss of type I pseudoexons are nearest to the causative de novo mutation. **5**: 50/52 match of bss to the 3ʹ or 5ʹ ss of pseudoexons type II (Figure 2(C)). 6: 48/50 type II pss match intron bss with top 3 reads. 7: 20/20 match between bss and 3ʹ recursive splice sites identified in a genome screen [42] **8**: 124/148 match between bss and 3ʹRS and 5ʹRS of DMD^43^ **9**:72/72 match between bss and aberrant ss activated by mutations of the spliceosome.

Figure 2 shows that background splice sites also strongly match de novo ss mutations, pseudoexon splice sites, recursive splice sites and the aberrant splice sites that are activated in cancer. These findings are discussed below and in Appendices 2 to 4.

## Discussion

Our analysis shows that when a splice site is mutated, the non-mutated partner splice site(s) instead usually splices to its strongest background splice sites. Consequently, the effect of splice site mutations upon css activation or exon skipping (Figure 1(A)) can be predicted from background splicing information for normal genes. Table 2 shows that css match bss in about 85% of cases and that 75% of 5ʹ css and 64% of 3ʹcss match bss with the most reads. When exon skipping only is caused by a splice site mutation this correlates with higher background reads for skipping compared to candidate css reads in 125/143 (87%) of examples (Table 3). Table 4 shows that the experimental reports of multiple exon skipping caused by splicing mutations also correlate well with background splicing reads. Consequently, an initial consideration of background splicing gives a useful indication of the primer design required to investigate the likely effect of a potential intron splice site mutation and should help to interpret RT-PCR results, particularly those that are complicated by alternative splicing (Appendix 1). It should be noted that this paper is not informative about intron retention, which is an aberrant effect that is sometimes caused by splice site mutations.

Background splice sites also strongly match the splice sites used for other aspects of splicing (Figure 2, Appendices 2 to 4 and see below) and background splicing information is also of value to splicing therapy (Appendix 5 and see below).

We generally restricted our css candidates to bss within 1000 bases of the mutated intron ss (Figure 1(A)), because this is a known feature of css activation [6] (Table S2 index). However, many intron bss are greater than 1000 bases from an intron ss and in about 10% of introns, these sites have the highest number of reads (Table S3). Some of these more distant bss have facilitated pseudoexon formation and some are recursive splice sites (see below).

Eight percent of css did not match bss listed in Snaptron but this value is likely to decrease as RNA sequencing databases increase in size (see below). There might however be a higher level of false positives, ie bss within 1000 bp of a splice site mutation that are not activated as css. For example, Table 1 lists four top bss of BRCA1 (column 5, rows 5, 24, 31, 35) that were not activated as css despite having more reads than the bss that matched the css. Of course some of these top bss might be identified as css in subsequent experiments, Figure 1B provides an example of this. The upper limit of top bss that are css false positives can be estimated from Table 2 as the proportion of css that matched bss that did not have the highest reads. For DBASS5 this is 51/201 (25%) and for DBASS3 35/97 (36%). Other methods give a lower false-positive estimate (Appendix 1).

For multiple exon skipping, we suggest that the level of false positives indicated by Table 4 (10 out of 42), is an upper limit. We included these ten examples because the RT-PCR primers that were used were capable of detecting the multiple exon skips indicated by Snaptron (Table 4). However, there may be other reasons why some of these skipping events, if they occurred, were not reported.

Six of the 199 mutations chosen from DBASS5 and 2 of 99 mutations from DBASS3 (Table S2) generate more complicated patterns of aberrant splicing than those illustrated in Figure 1A. These are separately analysed and discussed in Appendix 6.

Snaptron has four different RNA sequencing databases [9] (Materials and methods). We initially analysed the first database SRAv1 but as a control we also analysed BRCA1 and BRCA2 splicing mutations using the smaller GTEx and larger SRAv2 databases (Table S6). We found 35 experimentally reported css from both BRAC1 and BRCA2 of which 29 match bss listed in SRAv1 (Table S6). Use of the larger SRAv2 database increased the number of matches to 32/35, whereas the smaller GTEx database, which is made entirely from normal (non-diseased) tissue, had only 18/35 matches. Table S6 shows that the ratio of intron to css reads for each of the css of BRCA1 and BRCA2 have similar values when calculated from GTEx or from the SRA databases, so demonstrating that css usage occurs at similar frequencies in the three databases. Therefore, background splicing is a property of normal genes expressed in normal tissues, as expected [7,8].

The match between 5ʹcss and bss in DBASS increases from 201 to 219/237 (92%) with SRAv2 and from 97 to 101/110 (92%) for 3ʹcss (Tables 2, S2). However, the match between bss and css reports for DMD is less than average (Table 2). The match between css and bss increased slightly with the use of the larger SRAv2 database from 10/22 to 13/22, this is again below average most probably because there are still relatively few sequencing reads for DMD even in SRAv2 (Table S2).

Genetic disease is also caused by de novo splice site mutations (Table S2 index), which may also activate pseudoexons (Figure 2(A,B)). Snaptron shows that in 47/50 cases de novo ss match bss, which means that these sites were already active at a low level prior to the de novo mutations that enhanced already existing GT, GC or AG dinucleotides (Figure 2(D), Appendix 2). The filtering system of the Snaptron database precludes testing whether de novo GT, GC or AG splice sites matched bss prior to their creation (Appendix 2)

The 3ʹ pseudoexon splice sites (pss) that are co-activated by 5ʹ de novo splice sites (Figure 2(B)) matched bss in 63/71 cases and 14/22 for 5ʹpss (Figure 2(A, D)). Of these bss matches, 71/77 were the nearest bss to the de novo ss mutation (Figure 2(D), Appendix 2). For those pseudoexons (Figure 2(C)) that are generated typically by mutations of auxiliary splicing motifs we report that the pseudoexon splice sites match particularly active bss in 48 out of 52 examples (Figure 2(D) column 6, p = 1 × 10^−10^, Appendix 2). An analysis by Keegan [22] indicates that the splice sites of this type of pseudoexon (Figure 2(C)) often originate from recursive splice sites.

There are many excellent in silico programmes that can assess whether a variant of unknown significance is likely to generate a de novo splice site or to disrupt a splicing regulatory element [23,24]. In addition, it may prove useful to cross-reference the Snaptron database which provides information as to whether a candidate de novo ss was active prior to the mutation and whether candidate mutations of auxiliary splicing motifs lie within or are in close proximity to semi-dormant pseudoexons (Appendix 2).

There are a number of possible improvements that can be made to the method presented here. Systematically comparing the observed usage of bss with their splice site strength and presence of auxiliary splicing sites, as used by *in silico* modelling methods [2–5,23,24] may prove to be informative for both approaches. Expected future increases in size of the GTEx RNA sequencing database will facilitate the comparison of experimental data, which is often obtained from patient lymphocytes, with background splicing data from the most relevant normal tissue.

Sibley et al (2015) previously established that recursive splice sites and recursive exons can be identified from RNA seq data [25]. In agreement with this we found that the large majority of reported recursive ss match bss, particularly those bss with high reads (Figure 2(D), Appendix 3). Consequently, the Snaptron database contains a very large number of recursive ss candidates.

Mutations of the spliceosome are reported to activate novel aberrant splicing events in leukaemia and other cancers [26–31]. We report that 72/72 of the aberrant splice sites in cancer samples that we analysed match bss in RNA splicing databases made from normal tissue (Figure 2(D), Appendix 4). Furthermore, the bss that match the cancer ss have relatively high reads compared to other bss (Appendix 4). Our finding that mutations of the spliceosome enhance strong bss, rather than activate entirely novel ss, is consistent with the likely subtle effects of the spliceosome mutations upon splice site recognition [31,32] and is consistent with a previous report that 80% of exon–exon junctions that were thought to be cancer specific are found in non-cancer cells [33].

An important goal is to identify which of many aberrant splicing pathways have a causal role in cancer. There is strong evidence that mutations of splicing components SRSF2 and SF3B1 cause cancer in part by enhancing the inclusion of pseudoexons with in-frame stop codons for two genes EZH2 and BRD9, respectively, [34–36]. The ‘poisoned’ pseudoexon of EZH2 is conserved and expressed in healthy tissue [34]. Snaptron shows that the poisoned pseudoexon of BRD9 is also spliced in healthy tissue, at 5% of the level of the host intron (Appendix 4). Therefore in both causal cases the splicing machinery mutations enhance alternative splicing events that are arguably already established.

Antisense oligonucleotides (ASOs) are often used to correct mutations that create de novo splice sites and pseudoexons [37]. However, the use of ASOs to restore normal splicing by blocking css is rarely reported. We identified three such experimental reports after searching PubMed [38–40]. In all cases the target css are activated by relatively weak splice site mutations and the css originates from a dominant bss (Appendix 5). Table S2 (DBASSw) lists 44 medical syndromes that can be caused by weak 5ʹ or 3ʹ splice site mutations and in 25 of these cases the activated css matches a dominant bss (Appendix 5, Table S2), indicating that these 25 cases are also good candidates for the same approach.

ASOs that are designed to cause single exon skipping, sometimes cause double exon skipping as an unwanted side effect [41,42]. Background splicing information can identify likely double and multiple exon skipping events caused by splice site mutations (Table 4) and may also predict multiple skipping effects of ASOs (Appendix 5).

## Supplementary Material

Supplemental Material

## Data Availability

All data used here is available in the supplementary files except for the raw Snaptron data (for example Figure 1(B,C)), which can either be freely downloaded from the Snaptron database (see Appendix 1) or is available upon request.
