# Supplementary material for: Background splicing as a predictor of aberrant splicing in genetic disease
Source: RNA Biol. 2022 Feb 19;19(1):256–65. doi: 10.1080/15476286.2021.2024031 (PMC8865296; doi:10.1080/15476286.2021.2024031)
Supplement: Supplemental Material [file KRNB_A_2024031_SM7960.zip › Supplementary information/Appendix 3 recursive splicing.docx]

**Appendix 3. Recursive splicing**

Large introns are removed in sections by a process called recursive splicing that uses internal splice sites within introns (Burnette et al 2005; Sibley et al 2015; Kelly et al 2015; Gazzoli et al 2016). We analysed the first 20 of over 2000 recursive splice sites discovered by a screen of the human genome by Kelly et al 2015 and Tables A3 and S4 show that all of these sites matched background ss, as would be expected (Sibley et al 2016) and that in 12/20 cases the matching background ss had the highest reads of all bss within an individual intron. Similarly, Tables A3 and S4 show that 58/71 and 66/77 of 5’ and 3’ recursive splices identified in 36 human DMD introns by Gazzoli et al 2016 (21) matched background ss. The 3’ and 5’ recursive ss matched a background ss with the highest reads in 26 and 27 of the 36 introns (Table A3, S4).

Table A3 recursive splice sites

| Recursive splice sites | Snaptron match | Top rank |
| --- | --- | --- |
|  |  |  |
| Kelly et al 2015 3'RS | 20/20 | 12/20 |
| Gazzoli et al 2016 3'RS | 58/71 | 26/36 |
| Gazzoli et al 2016 5'RS | 66/77 | 27/36 |

Table A3. Recursive spice sites. Summary of a comparison of recursive ss identified by Kelly et al 2015 (19) and Gazzoli et al 2016 (21) with background ss listed in Snaptron (see Table S4). Column 2 shows that the first 20 recursive splice sites listed by Kelly et al 2015 matched background ss and also shows how many of the RS reported by Gazzoli et al 2016 matched background ss. Column 3 shows how many times recursive ss matched the background ss within an intron with the most reads. Further details (Table S4).

References

Burnette, J.M., Miyamoto-Sato, E., Schaub, M.A., Conklin, J. and Lopez, A.J. (2005) Subdivision of large introns in Drosophila by recursive splicing at nonexonic elements. *Genetics*, **170**, 661-674.

Kelly, S., Georgomanolis, T., Zirkel, A., Diermeier, S., O'Reilly, D., Murphy, S., Langst, G., Cook, P.R. and Papantonis, A. (2015) Splicing of many human genes involves sites embedded within introns. *Nucleic Acids Res*, **43**, 4721-4732.

Sibley, C.R., Emmett, W., Blazquez, L., Faro, A., Haberman, N., Briese, M., Trabzuni, D., Ryten, M., Weale, M.E., Hardy, J. *et al.* (2015) Recursive splicing in long vertebrate genes. *Nature*, **521**, 371-375.

Gazzoli, I., Pulyakhina, I., Verwey, N.E., Ariyurek, Y., Laros, J.F., t Hoen, P.A. and Aartsma-Rus, A. (2016) Non-sequential and multi-step splicing of the dystrophin transcript. *RNA Biol*, **13**, 290-305.
