# Supplementary material for: Background splicing as a predictor of aberrant splicing in genetic disease
Source: RNA Biol. 2022 Feb 19;19(1):256–65. doi: 10.1080/15476286.2021.2024031 (PMC8865296; doi:10.1080/15476286.2021.2024031)
Supplement: Supplemental Material [file KRNB_A_2024031_SM7960.zip › Supplementary information/Appendix 4 .docx]

**Appendix 4 – spliceosomal mutations**

Mutations of the spliceosome, in particular of SF3B1, are reported to activate novel aberrant splicing events in leukaemia and other cancer^26-32^. We compared the most novel of the css caused by SF3B1 and U1 mutations with the background splicing events in Snaptron SRAv1 (Table S5a). This analysis is summarised in Table A4-1 and it shows that 122/123 of the cancer aberrant splicing events matched background ss (Table A4-1 column C rows 1 to 7) with relatively high read numbers in Snaptron (Table A4-1, column D). A similar 72/72 match was obtained by analysis of Snaptron gtex, which is made from normal tissues (Table S5b)

Table A4-1-1. Spliceosome mutations and cancer

|  | A | B | C | D | E | F | G |
| --- | --- | --- | --- | --- | --- | --- | --- |
|  | Spliceosome mutation | Aberrant  splicing type | Match to  bss | Match to top 3 bss reads | Match to nearest  3 bss | Read ratio  (normal/  aberrant) | Source  Table S5a |
|  |  |  |  |  |  |  |  |
| 1 | SF3B1 | 3' css | 26/26 | 26/26 | 19/26 | 1334 | Sheet 2 |
| 2 | Darman et al 2015 | 5' css | 20/20 | 18/20 | 2/20 | 497 | Sheet 3 |
| 3 |  | exon skip | 20/20 | 17/20 | na | 39 | Sheet 4 |
| 4 |  | exon inclusion | 11/11 | 22/22 | na | 26 | Sheet 5 |
|  |  |  |  |  |  |  |  |
| 5 | SF3B1 | 3'css proximal | 10/10 | 10/10 | 9/10 | 376 | Sheet 6 |
| 6 | DeBoever et al 2015 | 3'css distal | 12/12 | 10/12 | 4/12 | 1900 | Sheet 7 |
| 7 | U1 | 5'css | 23/24 | 19/24 | 7/22 | 1504 | Sheet 9 |
|  | Suzuki et al 2019 |  |  |  |  |  |  |

Legend to Table A4-1-1. Aberrant splice sites in cancer match background ss. This table summarises Table S5a. Column A lists two of the spliceosomal components that are mutated in cancer and column B describes the aberrant splicing types reported by these authors. Column C shows the number of aberrant ss identified in the chosen cancer samples that match background ss from Snaptron. Column D shows how many times the cancer ss match background ss within the top 3 bss reads. Column E shows how often the 5’ and 3’css match background ss that are nearest to a 5’ or 3 intron ss. Column F shows the ratio of reads for normal intron splicing divided by the reads for the aberrant splicing event from Snaptron and column G shows which sheet of Table S5a is summarised by each row of Table A4-1.

The read numbers for the bss that match cancer css are in the order of 1000 fold less than the reads for normal intron removal, as shown in column F rows 1, 2, 5, 6 & 7 of Table A4-1. By contrast, the rarer exon skipping or exon inclusion events that are enhanced by SF3B1 mutations (Table A4-1 rows 3, 4) have background splicing reads only 39 or 26 fold lower on average than normal intronic splicing.

Mutations of the splicing components U2AF and SRSF2 are reported to cause quantitative rather than qualitative changes in splicing^29,30^, whereas mutations of the small non-coding RNA U1 are reported to activate novel splicing events in SHH medulloblastomas^31^. We analysed 20 css with the lowest false discovery rate values of over 1300 css reported by Suzuki et al^31^ plus aberrant splice sites for PTCH1, GLI2, CCND2 and PAX5, which are implicated in this cancer (Tables A4-1 row 7, S5a sheet 9). We found that 23 out of 24 of these css caused by U1 mutations matched background splice sites and that 19 out of the 24 css matched background ss with top three reads (Tables A4-1, S5a sheet 9).

There is strong evidence that mutations of splicing components SRSF2 and SF3B1 cause cancer in part by enhancing the inclusion of pseudoexons with in-frame stop codons for two genes EZH2 and BRD9 respectively ^34-36^. The ‘poisoned’ pseudoexon of EZH2 is conserved and expressed in healthy tissue ^34^.

Table A4-2

|  | Reads | Reads | Reads | 3'pss rank | 5'pss rank |
| --- | --- | --- | --- | --- | --- |
|  | 5'ss to 3'ss | 5'ss to 3'pss | 5'pss to 3'ss |  |  |
|  |  |  |  |  |  |
| EZH2 | 104841 | 28451 | 24998 | 1(7) | 1(2) |
| BRD9 | 412817 | 15748 | 27680 | 2(15) | 1(17) |

Legend to Table A4-2. Sequencing reads for the host introns (column 1) and poisoned pseudoexons (columns 2, 3) of the genes EZH2 and BRD9 - from the Snpatron GTEx spliced RNA database. Extract from Table S5a worksheet 10.

Snaptron confirms that the pseudosplice sites of the poisoned pseudoexon of EZH2 are spliced in normal tissue - at about 25% of the frequency of the intron ss of the host intron (Table A4-2). This table also shows that the pseudoexon of BRD9 is spliced in healthy tissue, at 5% of the level of the host intron. Columns 4 and 5 show that the pseudoexon splice sites are amongst the most active of the ss within the host intron.
