# Supplementary material for: Background splicing as a predictor of aberrant splicing in genetic disease
Source: RNA Biol. 2022 Feb 19;19(1):256–65. doi: 10.1080/15476286.2021.2024031 (PMC8865296; doi:10.1080/15476286.2021.2024031)
Supplement: Supplemental Material [file KRNB_A_2024031_SM7960.zip › Supplementary information/Appendix 5 splicing therapies (1).docx]

**Appendix 5 - splicing therapy and background splicing**

It has long been established that antisense oligonucleotides (ASOs) can correct mutations that create de novo splice sites and pseudoexons (Dominski & Kole, 1993). However, the use of ASOs to block css that are activated by mutations of splice sites are rarely reported. We identified three such experimental reports after systematically searching PubMed with the term: cryptic splice site splicing therapy (Hu et al 2019; Oustric et al 2014; Uchimaya, 2007). In all cases the css are activated by relatively weak splice site mutations and the css originates from a dominant bss (see below). There are other examples from Table S2 (DBSSw) that fit this pattern and are therefore good candidates for the same approach.

Hu et al (2019) describe an ASO against an activated 3’css of the gene CAPN for the possible treatment of limb-girdle muscular dystrophy type 2A. Panel A of Fig A5-1 illustrates that the ASO blocks a css at -398 that is activated by deletion of a single T base 29 bases downstream from the acceptor cleavage site of intron 6 of CAPN3. ASO treatment results in the restoration of normal intron removal plus a mild side effect of weak activation of a css at -51 (Fig A5-1A). The Snaptron database (Fig A5-1B) contributes the information that the css at -389 has by far the most reads (1701) of any background ss within this intron and that the css at -51 has 11 reads and is one of several candidate css with relatively low reads compared to css -389 (Fig A5-1, B). Css -389 also has considerably more background reads than exon 7 skipping with 115 reads (Fig A5-1, B).

**Figure A5-1**

A


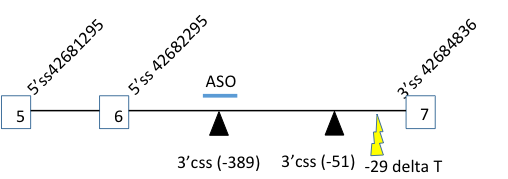


B


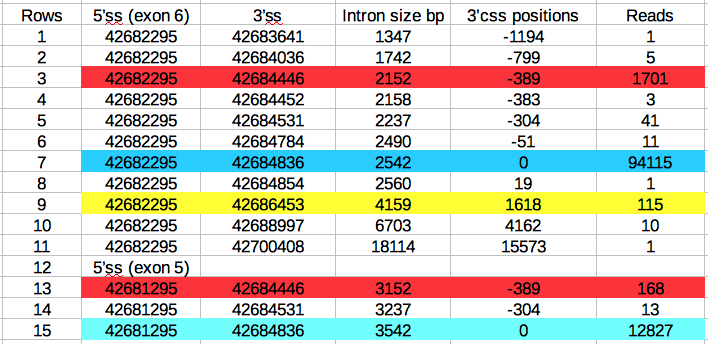


Figure A5-1. A. Exons 5 to 7 of the CAPN3 gene. Hu et al 2019 report that deletion of a thymidine 29 bases upstream from the 3’ss at the start of exon 7 (42684836 hg19) strongly activates a css at -389. Exon 7 is alternatively spliced to exons 5 and 6 and the mutation caused the insertion of 389 bases into the major exon product 5,6,7 and into the minor exon product 5,7. An antisense oligo against css -389 restored normal splicing apart from the insertion of 51 bases into a small percentage of exon product 5,6,7 but not 5,7. Panel B shows the background and normal splicing reads for the 5’ss of exons 5 and 6 of the wild type CAPN3 gene (srav1). The row in darkest blue shows the reads for splicing between exons 6 and 7 and the lighter blue row shows the reads for splicing between exons 5 and 7. Red rows show reads between exons 5 or 6 with the 3’css -389 and the yellow row shows reads for downstream single exon skipping of exon 7.

Oustric et al (2014) report an ASO that can largely restore normal splicing of the gene FECH following a T to C mutation 48 bases upstream from the 3’ss of intron 3 that activates a css at -63 bp. The ASO anneals from -63 to -45 bp and so covers both the css and the causative mutation. The Snaptron database (see below) shows that the 3’css at -63 bp (shaded red) is a strong bss that has far more splicing reads (7174) with the 5’ss of exon 3 of FECH than any other bss. Reads for normal splicing are shown in blue and background single exon skipping in yellow.


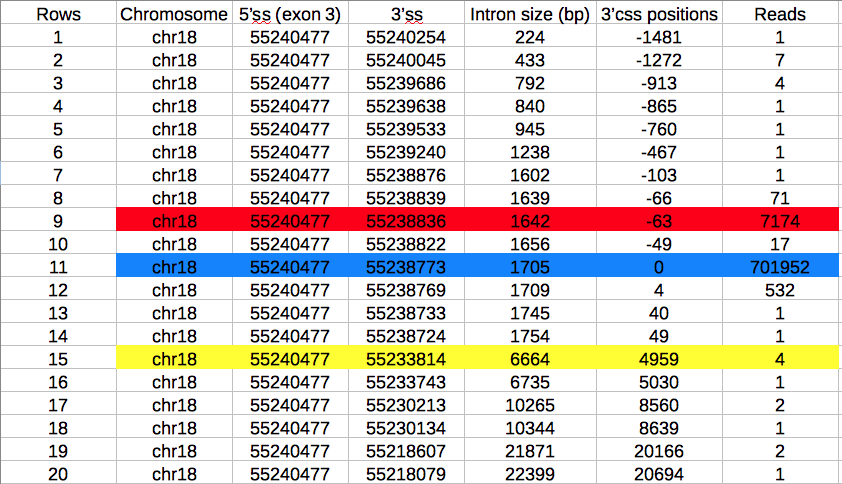


Table A5-1. Background and normal splicing reads involving the 5’ss of exon 3 of FECH (from Snaptron SRAv1, hg19). See text for further details.

Uchikawa et al (2007) demonstrated that an ASO against 5’css at positions +65 and +69 of BRCA-1 (Fig 1B) could partly restore the normal splicing that is disrupted by the mutation +6T>C of the 5’ss of intron 16 (Scholl et al 1999). The background splicing reads for these css are considerably higher than the reads for other 5’css candidates and for exon skipping (Fig 1B).

In all three cases described above the relatively weak splice site mutations do not prevent the restoration of some level of normal splicing following ASO treatment, as anticipated by the authors (Uchikawa et al 2007; Oustric et al 2014; Hu et al 2019). The low reads for other background splicing events indicates why blocking the activated css did not lead to a strong activation of a different css or of exon skipping (Sadusky et al 2004, Balestra et al 2015).

Uchikawa et al (2007) also examined a G to A mutation of the last base of exon 3 of PTCH1 that activates an intronic css at +37 (Nagoa et al 2005). An ASO against the css partly restores normal splicing in a minigel system. Snaptron has 91 reads for the css at +37 (Table A5-2 red shading) but it also has 48 reads for a single exon skip (yellow shading).

Table A5-2

| Rows | 5’ss | 3’ss (exon 4) | Intron size (bp) | 5’bss positions | Reads |
| --- | --- | --- | --- | --- | --- |
| 1 | 98602476 | 98244486 | 357991 | -354510 | 1 |
| 2 | 98350536 | 98244486 | 106051 | -102570 | 1 |
| 3 | 98278750 | 98244486 | 34265 | -30784 | 19 |
| 4 | 98270442 | 98244486 | 25957 | -22476 | 5 |
| 5 | 98268688 | 98244486 | 24203 | -20722 | 48 |
| 6 | 98268075 | 98244486 | 23590 | -20109 | 1 |
| 7 | 98255230 | 98244486 | 10745 | -7264 | 1 |
| 8 | 98247966 | 98244486 | 3481 | 0 | 79488 |
| 9 | 98247961 | 98244486 | 3476 | 5 | 3 |
| 10 | 98247929 | 98244486 | 3444 | 37 | 91 |
| 11 | 98246595 | 98244486 | 2110 | 1371 | 1 |
| 12 | 98245794 | 98244486 | 1309 | 2172 | 2 |
| 13 | 98244880 | 98244486 | 395 | 3086 | 1 |

Table A5-2. Background and normal splicing reads involving the 3’ss of exon 4 of PTCH1 (from Snaptron SRAv1, hg19). See text for further details.

The authors showed that blocking the +37 css with an ASO or reverting it restored normal splicing and did not result in the activation of a secondary css, which is consistent with the very low background reads for other css candidates. This PTCH1 5’ss mutation was not reported to activate single exon skipping (Nagao et al 2005) and exon skipping was not tested in the ASO experiment (Uchikawa et al., 2007). However, exon skipping in response to the ASO is a possibility given the relatively high background reads for this event (Table A5-2) and recent reports of such secondary activation events by ASOs (Hu et al. 2019; Balestra et al 2015).

Table S2 (DBSSw) lists examples of inactivating 5’ and 3’ss mutations that lie outside the core ss region. For 25 of these 44 cases the splice site mutation activates a css with considerably more background reads than the reads for the next best css candidate or for exon skipping (compare columns O, P and R of Table S2 DBSSw). These examples may also be amenable to the approaches described above, particularly as these weak splice site mutations may only achieve a phenotypic effect by enhancing relatively active bss.

Some of the examples of Table S2 (DBSSw) report multiple effects of the splice site mutation yet are still candidates for splicing therapy. For example the 3’ss mutation of the gene GAA causes glycogen storage disease and activates both exon skipping and a css at +518, in exact agreement with the Snaptron data (Table S2 DBSS3w). In this case normal splicing can be partly restored by targeting the causative mutation (Aung-Htut et al 2020).

Antisense oligonucleotides have also been developed to induce single exon skipping for the treatment of Duchenne muscular dystrophy (Matsuo 1996; Aartsma-Rus et al 2005). Wilton et al (2007) have categorised individual DMD exons according to the ease with which they can be skipped, with category 4 exons as the most difficult. We wondered whether single exon skipping is favoured when background reads for this event outnumber reads for competing background splicing events involving the same exon (Fig A5-2).

Figure A5-2

A


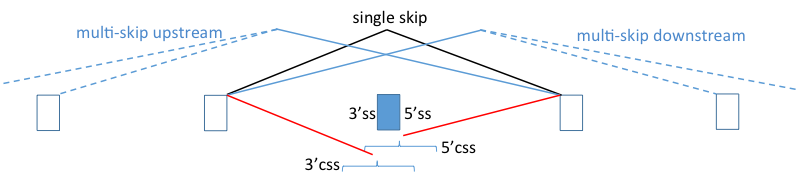


B


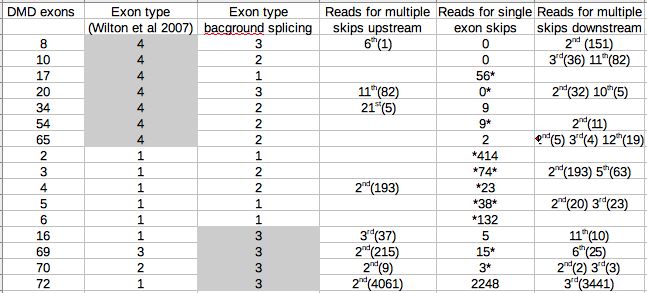


Figure A5-2. A. Diagram of background splicing information of possible relevance to single exon skipping by antisense oligonucleotides. Normal intron splicing is not shown. The exon in blue represents an exon that is a target for skipping by ASOs and the various lines between flanking exons illustrate background splicing of possible relevance to this aim. The black lines illustrate background reads for single exon skipping, the red lines background reads for potential css (background ss within 1000 bp of an intron ss) and the blue lines represent multi-exon skipping events, upstream or downstream. Panel B. Column 1 is a selection of DMD exons from Table S7 and column 2 shows their ease of skipping by ASOs, where 1 is highly efficient and 4 is inefficient (Wilton et al 2007). Column 3 shows our theoretical classification of the ease of exon skipping, where 1 is efficient and 3 is inefficient. The numbering in column 3 is decided by comparing the background splicing reads for single exon skips (column 5) with the upstream and downstream multiple splicing events involving the same exon (columns 4 and 6) and see Fig A5-2A. For example exon 20 is classified as 3 (column 3) because the reads for background single exon skipping are less than competing background reads both upstream and downstream. Asterisks indicate that the effect of mutations of one or both splice sites of these exons are known (see Table S7 for further details).

These competing background splicing events include multiple exon skipping, either upstream or downstream of the exon target, or both. The activation of css by ASOs for DMD is a rare occurrence (Aartsma-Rus et al 2005; Wilton et al 2007), perhaps because ASOs that block exon inclusion also block the recognition of nearby css. We therefore ignored the possible activation of css by ASOs as a factor that might compromise exon skipping by ASOs. Fig A5-2B compares all of the category 4 exons and the first five category 1 exons (Wilton et al., 2007) with the background reads for single and multiple exon skipping. We also included the remainder of the exons that we would predict to be the most difficult to skip, as indicated by their background splicing reads.

As can be seen (Fig A5-2B) there is not a strong overlap between the experimental results and our predictions, although there are points of agreement. Targeting of exons 8 and 54 with ASOs is found experimentally to cause the skipping of both exons 8 and 9 and of both exons 54 and 55 (Wilton et al 2007). This agrees with the background splicing data which has higher reads for downstream double exon skips than for single skips of exons 8 and 54 (Fig A5-2B). Targeting of exon 10 caused multiple but variable downstream exon skipping (Wilton et al 2007), which is consistent with the dominant background reads for multiple downstream skipping of this exon (Fig A5-2B). Aartsma-Rus et al (2005) reported downstream double exon skipping with ASOs against exons 8, 40, 58, 71, 73 and 77, which is also a good fit to the Snaptron data (Table S7). However, there are 9 further downstream double exon skips indicated by Snaptron (exons 3, 14, 20, 28, 54, 56, 58, 65, 75) that have not been reported (Table S7).

There is an average of 10, 85, 801 and 981 background reads for single exon skips for the exons categorized as 4, 3, 2 and 1 (Wilton et al 2007, Table S7, column O). This indicates that exons with high background reads are easier to skip with ASOs, however, this cannot be the only factor because five of the easy category 1 exons (Wilton et al 2007) have no background reads for single exon skipping (Table S7).

Perhaps more importantly, Fig A5-2B illustrates that some DMD exons have more background reads for skips of 3, 6, 10, 11 and 12 exons than for single exon skips. There are also examples of highest background splicing reads for 3, 4 or 5 skips of exons from other genes listed in Table S3. Some of the background reads for multiple exon skipping in DMD do not increase in number between SRAv1 and the larger SRAv2 spliced RNA databases (Table S7), which is of concern. However, in general, background splicing information is reasonably predictive of multiple skipping in response to splice site mutations (Table 4). As discussed above there is some degree of correspondence between reports of double exon skipping caused by ASOs and background splicing information, which suggests that it might be important to test for larger scale skipping predictions.

**References**

Aartsma-Rus, A., De Winter, C.L., Janson, A.A., Kaman, W.E., Van Ommen, G.J., Den Dunnen, J.T. and Van Deutekom, J.C. (2005) Functional analysis of 114 exon-internal AONs for targeted DMD exon skipping: indication for steric hindrance of SR protein binding sites. *Oligonucleotides*, **15**, 284-297.

Aung-Htut MT, Ham KA, Tchan M, Johnsen R, Schnell FJ, Fletcher S, Wilton (2020) Splice modulating antisens oligonucleotides restore some acid-alpha-glucosidase activity in cells derived from patients with late onset Pompe disease. SD.Sci Rep. Apr 21;10(1):6702

Balestra et al (2015) Regulation of a strong F9 cryptic 5'ss by intrinsic elements and by combination of tailored U1snRNAs with antisense oligonucleotides. *Hum Mol Genet*, **24**, 4809-4816.

Dominski Z and Kole R (1993). Restoration of correct splicing in thalassemic pre-mRNA by antisense oligonucleotides. Proc. Natl. Acad. Sci USA **90**, 8673-8677

Hu, Y., Mohassel, P., Donkervoort, S., Yun, P., Bolduc, V., Ezzo, D., Dastgir, J., Marshall, J.L., Lek, M., MacArthur, D.G. *et al.* 2019 Identification of a Novel Deep Intronic Mutation in *CAPN3* Presenting a Promising Target for Therapeutic Splice Modulation (2019). J Neuromuscul Dis. 2019 ; 6(4): 475–483.

Matsuo, M. (1996) Duchenne/Becker muscular dystrophy: from molecular diagnosis to gene therapy. *Brain Dev*, **18**, 167-172

Nagao K, Togawa N, Fujii K, Uchikawa H, Kohno Y, Yamada M, Miyashita T (2005) Detecting tissue-specific alternative splicing and disease-associated aberrant splicing of the PTCH gene with exon junction microarrays. Hum Mol Genet 14:3379–3388

Oustric V., Manceau H., Ducamp S., Soaid R., Karim Z., Schmitt C., Mirmiran A., Peoc’h K., Grandchamp B., Beaumont C. Lyoumi S, Moreau-Gaudry F, Guyonnet-Dupérat V, de Verneuil H, Marie J, Puy H, Deybach JC, Gouya L (2014)

Antisense oligonucleotide-based therapy in human erythropoietic protoporphyria. Am. J. Hum. Genet. 94:611–617.

Sadusky, T., Newman, A.J. and Dibb, N.J. (2004) Exon junction sequences as cryptic splice sites: implications for intron origin. *Curr Biol*, **14**, 505-509.

Scholl T, Pyne MT, Russo D, Ward BE. (1999) [BRCA5-1 IVS16+6T-->C is a deleterious mutation that creates an aberrant transcript by activating a cryptic splice donor site.](https://pubmed.ncbi.nlm.nih.gov/10406662/) Am J Med Genet.;85(2):113-6.

Uchikawa et al 2007. U7 snRNA-mediated correction of aberrant splicing caused by activation of cryptic splice sites. J Hum Genet 52:891–897 DOI 10.1007/s10038-007-0192-8

Wilton, S.D., Fall, A.M., Harding, P.L., McClorey, G., Coleman, C. and Fletcher, S. (2007) Antisense oligonucleotide-induced exon skipping across the human dystrophin gene transcript. *Mol Ther*, **15**, 1288-1296.
