# Supplementary material for: Background splicing as a predictor of aberrant splicing in genetic disease
Source: RNA Biol. 2022 Feb 19;19(1):256–65. doi: 10.1080/15476286.2021.2024031 (PMC8865296; doi:10.1080/15476286.2021.2024031)
Supplement: Supplemental Material [file KRNB_A_2024031_SM7960.zip › Supplementary information/Appendix_1_protocol.docx]

**Appendix 1. Protocol for using Snaptron splicing data to predict the effect of splice site mutations.**

BRCA1 splicing data was downloaded from Snaptron by using the link (http://snaptron.cs.jhu.edu/srav1/snaptron?regions=BRCA1). RNA splicing data for any other gene can be obtained by changing BRCA1 to the required gene name ie (http://snaptron.cs.jhu.edu/srav1/snaptron?regions=DMD). To access the other spliced RNA databases of Snaptron srav1 can be changed to srav2, gtex or tcga. Downloaded Snaptron data was then selected, copied and pasted into the spreadsheet LibreOffice Calc. We chose paste special, unformatted text, then UT-16 and tab options. The spreadsheet lists over 6000 differently spliced transcripts of BRCA1, although the large majority of these are background splicing events that are only supported by very low reads. Spreadsheet 1 was copied to two further worksheets. For sheet three we re-ordered the data by selecting the highest sequencing reads in column O (with the extended data option). We then chose and copied the top 30 rows with the highest sequencing reads into sheet 4 (the more rows that are chosen from sheet 3 the greater the number of minor alternative splicing events that can be seen). An edited version of sheet 4 is shown below (Table A1).

Table A1

|  | C | E | D | F |  | O |
| --- | --- | --- | --- | --- | --- | --- |
| Row | Chr | 5’ss | 3’ss | Intron | exon size | Reads |
|  |  |  |  | size |  |  |
| 1 | chr17 | 41277293 | 41276133 | 1161 |  | 30252 |
| 2 | chr17 | 41277287 | 41276133 | 1155 |  | 34608 |
| 3 | chr17 | 41276753 | 41276133 | 621 |  | 17172 |
| 4 | chr17 | 41276033 | 41267797 | 8237 | 99 (2) | 112381 |
| 5 | chr17 | 41267742 | 41258551 | 9192 | 54 (3) | 108919 |
| 6 | chr17 | 41258494 | 41256974 | 1521 |  | 7375 |
| 7 | chr17 | 41258472 | 41256974 | 1499 | 78 (5) | 88674 |
| 8 | chr17 | 41256884 | 41256279 | 606 | 89 (6) | 111753 |
| 9 | chr17 | 41256138 | 41251898 | 4241 | 140 (7) | 97874 |
| 10 | chr17 | 41256138 | 41251895 | 4244 |  | 34012 |
| 11 | chr17 | 41251791 | 41249307 | 2485 | 106 (8) | 109406 |
| 12 | chr17 | 41251791 | 41246878 | 4914 |  | 21537 |
| 13 | chr17 | 41249260 | 41247940 | 1321 | 46 (9) | 94639 |
| 14 | chr17 | 41247862 | 41246878 | 985 | 77 (10) | 123372 |
| 15 | chr17 | 41246760 | 41243050 | 3711 |  | 20462 |
| 16 | chr17 | 41243451 | 41243050 | 402 | 3426 (11) | 149785 |
| 17 | chr17 | 41242960 | 41234593 | 8368 | 89 (12) | 151941 |
| 18 | chr17 | 41242960 | 41234590 | 8371 |  | 14957 |
| 19 | chr17 | 41234420 | 41228632 | 5789 | 172 (13) | 106578 |
| 20 | chr17 | 41234420 | 41228629 | 5792 |  | 37608 |
| 21 | chr17 | 41228504 | 41226539 | 1966 | 127 (14) | 137477 |
| 22 | chr17 | 41226347 | 41223256 | 3092 | 191 (15) | 184837 |
| 23 | chr17 | 41222944 | 41219713 | 3232 | 311 (16) | 148299 |
| 24 | chr17 | 41219624 | 41215969 | 3656 | 88 (17) | 180029 |
| 25 | chr17 | 41215890 | 41215391 | 500 | 78 (18) | 180038 |
| 26 | chr17 | 41215349 | 41209153 | 6197 | 41 (19) | 182438 |
| 27 | chr17 | 41209068 | 41203135 | 5934 | 84 (20) | 153347 |
| 28 | chr17 | 41203079 | 41201212 | 1868 | 55 (21) | 173560 |
| 29 | chr17 | 41201137 | 41199721 | 1417 | 74 (22) | 144285 |
| 30 | chr17 | 41199659 | 41197820 | 1840 | 61 (23) | 137637 |

Table A1. Identification of the intron and major alternative splice sites of BRCA1 from a Snaptron download (see text). This is an edited version of an SRAv1 BRCA1 download from Snaptron, the column lettering refers to the original Snaptron download. The original columns D and E have been reversed (see text), most original columns have been deleted. The top 30 BRCA1 spliced sequencing reads (column O) were chosen and then the 5’ss coordinates in column E were reordered from high to low in order to show the introns and exons in a 5’ to 3’ direction. The 5’ss number is the first base of the intron and the 3’ss number is the last (hg19). Shading (columns E and D) shows alternative splice sites. Intron sizes are listed in column F. The exon size column was generated by us from columns E and D by using the constitutive splice sites and the 5’ or 3’ alternative splice sites with the most reads. Exons that are generated by alternative splicing are shaded, exon numbers are in brackets. The start codon of BRCA1 is located within the 99 bp exon 2 at position 31276103 and the stop codon is at position 41197697 within exon 24 (not shown). Exon 4 of BRCA1 is called exon 5 for historical reasons, the exon numbering shown is from the LOVD database (see materials and methods).

The purpose of sheet 4 is to identify the major constitutive intron splice sites and alternative splice sites. This helps to identify the background exon skipping events highlighted in yellow in Figs 1B,C and it also indicates whether additional analyses are required for cases complicated by alternative splicing (see below). The major alternative splice sites can easily be identified in Table A1 (shaded).

In sheet 4 (see Table A1) it is important to take note of the gene direction along the chromosome indicated by – or + in column G (strand). If + this means that the 5’ss are listed in column D (and 3’ss in column E) however, if column G is - then the 5’ss are listed in column E and the 3’ss in column D. BRCA1 is encoded on the – strand, which is why the order of columns D and E is reversed in Table A1.

Worksheets 1 and 2 were used separately to order the large numbers of 5’ss or 3’ss from Snaptron in a 5’ to 3’ direction. By this means 5’ or 3’ intronic ss and major alternative ss can be identified from sheet 4 (see Table A1) and all of their partner background ss can be identified from either sheet 1 or from sheet 2 (see Figure 1B,C). Occasionally, column G of worksheet 4 contained rows with both – and + values, due to overlapping transcripts from both strands. The required transcript usually has the greatest number of reads and can also be identified from the UCSC genome browser.

It is also useful to identify all of the partners of the mutated splice site at the start of the analysis in case this site is normally involved in alternative splicing. Major alternative splicing can be seen in sheet 4 of the spreadsheet and minor alternative splicing can identified by looking at the read numbers for the partner sites of the mutated splice site (in sheet 1 or 2). If there is no or little alternative splicing the analysis proceeds as outlined in Figure 1. If there are also alternative splice sites then these should be analysed alongside the main partner ss (see below) because these alternative splice sites may also participate significantly in aberrant splicing events. A splicing mutation of LAMP2 is a good example of this.

LAMP2

Table S2 DBASS5 Index row 177

Di Blasi, C., Jarre, L., Blasevich, F., Dassi, P. and Mora, M. (2008) Danon disease: a novel LAMP2 mutation affecting the pre-mRNA splicing and causing aberrant transcripts and partial protein expression. *Neuromuscul Disord*, **18**, 962-966.

Exons 5 to 9 of LAMP2

Exon 9A

Exon 5

Exon 6

Exon 7

Exon 85’ss

Exon 9B

Exon 9C

The authors (17) report the effect a mutation of the 5’ss of exon 8 of LAMP2 (119575584 hg19) that is normally involved in alternative splicing, as illustrated above. The use of alternative exons 9A, 9B or 9C generates three protein isoforms (LAMP2B, LAMP2A and LAMP2C). LAMP2 illustrates the importance of first checking whether the mutated ss is involved in alternative splicing. This is done by looking for all of the splicing partners of the mutated site (119575584) as illustrated below.


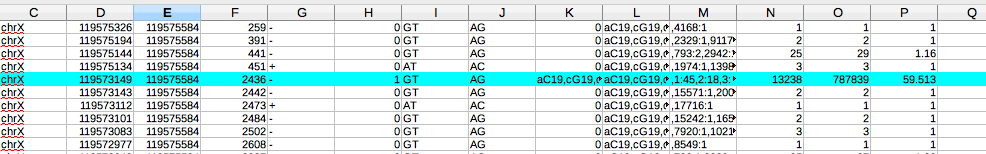


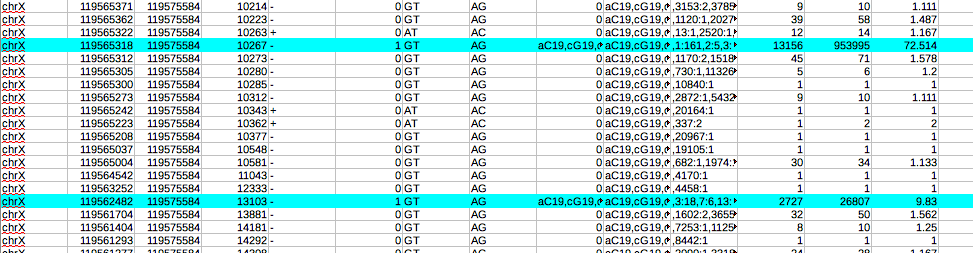


The 5’ss is listed in column E above because the gene is on the – strand, therefore the rows marked with a + in column G can be ignored.

Column O shows that the 5’ss has large numbers of reads with the 3’ass 119573149 and 119565318 and a smaller number of reads with the more minor 3’ass 119562482 (in blue). Mutation of the common 5’ss partner will therefore affect the splicing of all three sites, each of which therefore require analysis.


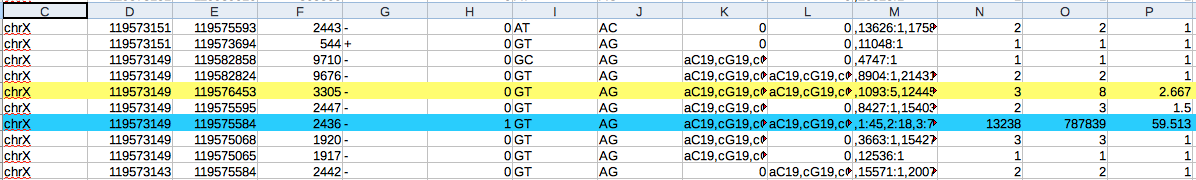


The above shows that the 3’ass 119573149 has most background reads (8, column O) for a single exon skip.


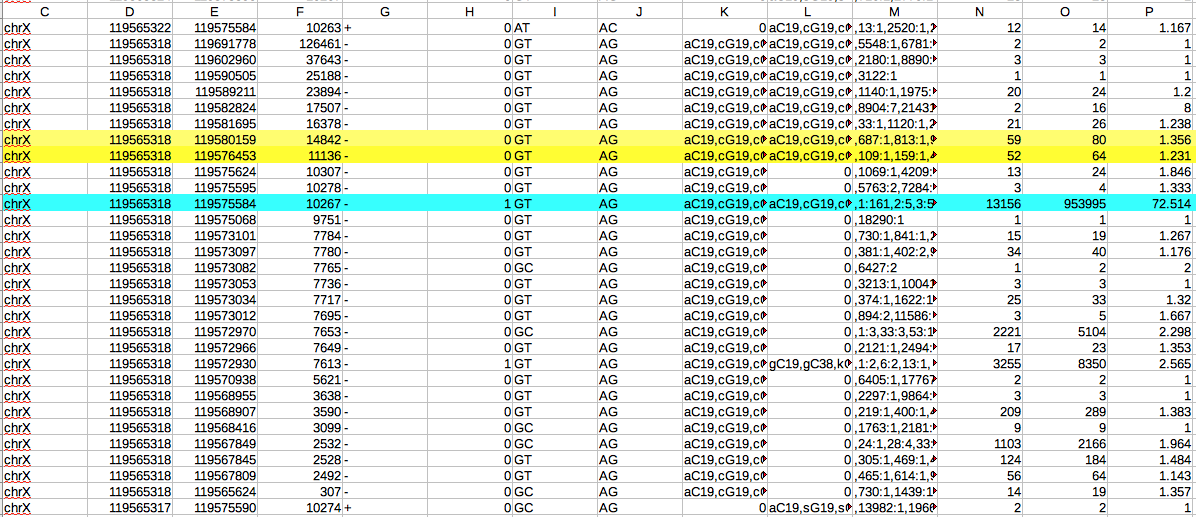


The above shows that the 3’ass 119565318 has similar reads for a double exon skip (80) and a single exon skip (64, column O). Note: that the much larger background reads of 5104 and 8350 etc (column O) are greater than 1000 bases away and are within the intron. Sites this far from the mutated 5’ss are rarely used as 5’css but might be recursive splice sites (see text).


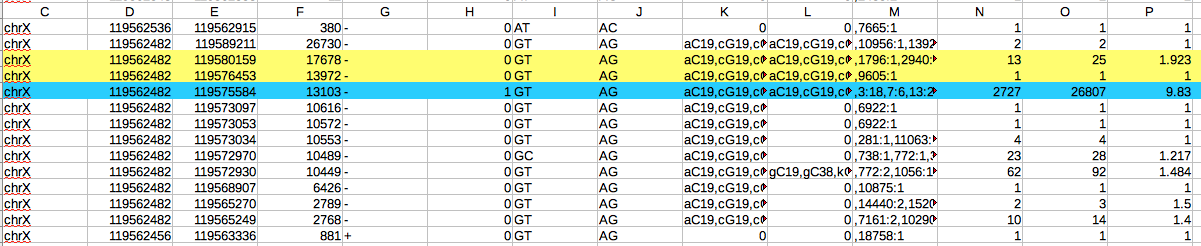


The above shows that the minor 3’ass has most reads (25, column O) for the double exon skip. Overall, the Snaptron reads compare very well with the author’s experimental reports about the effect of the 5’ss mutation upon exon skipping for the three LAMP2 isoforms (see Table 4).

Further notes:

We largely analysed background splice sites that splice to intron 5’ or 3’ ss (Figure 1A). Consequently background splicing events within introns and exons and across intronic ss were not usually considered, in order to focus upon the most relevant background splicing as illustrated in Fig 1A. We rescreened the 15% or so of experimentally identified css from DBASS for which we found no match to a background ss (Table 2) without this restriction and only found one clear example that we had missed by our approach (Table S2 5’css PKP1).

**False negatives and false positives**

The level of false negatives is quite low (see Discussion), which is reflective of the large number of bss that have been identified by sequencing, with the exception of genes that have very low levels of expression. There may however be bss within 1000 bp of a splice site mutation that despite having the most reads are not activated as css (false positives). At least two out of 6 possible false positives for BRCA2 (Table S2) are not reported as css despite repeated analysis.

The upper limit of top bss reads that might be false positives can be estimated from Table 2 as the proportion of css that matched bss that did not have the highest reads. For DBASS5 this is 51/201 (25%) and for DBASS3 35/97 (36%). Table S2 (css) column T gives all of the details and also indicates those non-matching bss with markedly higher reads than the bss that match css. This gives a false positive estimate of 22 out of 201 (11%) for DBASS5 and 19 out of 97 (20%) for DBASS3. An estimate of the level of possible false positives can also be made from Table 3 where 8 out of 79 reports (10%) of 5’ exon skipping only (from the 5’ skip database), nevertheless have higher background reads for candidate 5’ css than for the exon skips (for example Table 1 row 9). Similarly there are 10 out of 64 reports (16%) from the 3’ skip database of likely false positive 3’css candidates (Table 3).
