# Supplementary material for: Background splicing as a predictor of aberrant splicing in genetic disease
Source: RNA Biol. 2022 Feb 19;19(1):256–65. doi: 10.1080/15476286.2021.2024031 (PMC8865296; doi:10.1080/15476286.2021.2024031)
Supplement: Supplemental Material [file KRNB_A_2024031_SM7960.zip › Supplementary information/Appendix_2_de_novo_ss.docx]

**Appendix 2. De novo ss and pseudoexons**

Table A2-1 shows that de novo ss are the second largest mutation class in DBASS that causes human genetic disease after css.

Table A2-1

DBASS5 DBASS3

Css 459 182

Css (unusual) 14 39

De novo ss (created) 34 123

De novo ss (enhanced) 95 10

Pseudoexon activation 71 10

Pseudoexon activation (unusual ) 14 6

Table A2-1. Summary of the different types of splicing mutations in DBASS. DBASS lists mutations from a wide variety of human genes that cause disease by disrupting splicing (6). In DBASS5 there are 459 examples of inactivating mutations of 5’ss that activate 5’css, mainly within 1000 bases of the mutation (Table S2 index). There are also 14 examples of css that are activated by less common mutations (described here as unusual) that lie outside the nine bases of the 5’ss consensus sequence CAG/guragu. Similarly, for DBASS3 there are 182 examples of inactivating mutations of the 3’ss that activate 3’css and 39 examples of 3’ css that are activated by mutations outside the 3’ss consensus sequence yyycag/G. De novo ss are the second largest category of splicing defects, these splice sites are generated directly by the mutation. Pseudoexon activation is caused by mutations that generate a de novo 5’ss (DBASS5) or de novo 3’ss (DBASS3) and a pseudoexon is produced when this is also accompanied by the activation of a partner splice site (Fig 2A,B). Unusual pseudoexon activation refers to the creation of a pseudoexon by a mutation that does not create a de novo ss and usually lies within the pseudoexon (see Figure 2C).

De novo ss mutations can exist on their own or they may also activate pseudoexons as illustrated in Fig 2. In Table A2-1 (above) we divided the de novo mutations into two types, created or enhanced. Created refers to a mutation that creates the GT or GC dinucleotides of a 5’ de novo ss or that creates the AG dinucleotide of a 3’ de novo ss. Enhanced refers to mutations that enhance already existing GT, GC or AG dinucleotides. As expected none of the 34 and 123 created de novo ss of DBASS5 or DBASS3 match bss in Snaptron (Table A2-2, row1). Even if there were reads for the original dinucleotide these would have been filtered from this database as all Snaptron splice sites match canonical ss sequences (see Materials and methods).

Table A2-2. Background splice site matches to de novo and pseudo splice sites

| Rows | Type of mutation  or pseudoexon | Match to background splice sites in SRAv2 | | | |
| --- | --- | --- | --- | --- | --- |
|  |  | De novo ss  (created) | De novo ss  enhanced | 3’ pss | 5’ pss |
|  |  |  |  |  |  |
| 1 | 5’ de novo ss mutation | 0 (34) | 37 (40) |  |  |
| 2 | 3’ de novo ss mutation | 0 (123) | 10 (10) |  |  |
| 3 | Pseudoexon (Fig 2B) | 0 (36) | 31 (35) | 63(71) |  |
| 4 | Pseudoexon (Fig 2A) | 0 (20) | 1 (2) |  | 14(22) |
| 5 | Pseudoexon (Fig 2C) |  |  | 25 (26) | 25 (26) |

Table A2-2 legend. De novo splice sites and pseudoexons . Rows 1 to 5 summarise the information in Table S2 sheets 1 to 5. De novo splice site mutations were chosen systematically from DBASS for analysis (see Table S2 index). All of the pseudoexons reports in DBASS were analysed plus some additional pseudoexon reports from Dhir & Buratti 2010 and Vaz-Drago et al 2017 (Table S2).

There are 95 reports of mutations that enhance de novo ss in DBASS5 (Table A2-1) and we analysed the first 40 medical syndromes caused by this mutation type and report that 37 of these de novo ss positions exactly match bss from Snaptron (Table A2-2, row 1, Table S4). Similar results were found for mutations that generated 3’ de novo ss (Table A2-2, row 2).

The bss match to enhanced 5’ or 3’ de novo ss was found by using an unrestricted screen, by which we mean searching Snaptron for background splice reads for the genome reference number of the de novo splice site to any opposite splice site and not just to an intron splice site.

Pseudoexons are most commonly generated when a mutation that creates a 5’ or 3’ de novo ss also co-activates a partner pseudoexon ss (Fig 2A,B). The 5’ and 3’ de novo ss that initiate pseudoexon formation matched bss at a similar level to the de novo mutations only (Table A2-2 compare rows 1 with 3 and 2 with 4). For the 3’ pss that partner the 5’ de novo mutations, there is a match of 63 out of seventy one 3’pss with bss (Table A2, row 3) and for 5’pss a match of 14/22, these matches were made using the larger SRAv2 database. Of the 77 bss that matched pss, 71 were the nearest bss to the de novo mutations (Fig 2A,B, Table S4)

Pseudoexons that were created by means other than de novo ss mutations (Fig S2C) had the best match to bss (Table A2-2 row 5, Fig 2C). Twenty five out of 26 pairs of these pseudo splice sites matched background ss in Snaptron and notably 48 of these 50 pss matched bss with the top three reads within the intron in which the pseudoexon was formed (p = 1 x 10^-10^ , Table S4 sheet 5). Possibly the causative mutations are relatively weak and consequently may only have a phenotypic effect through enhancement of relatively active bss that together form a semi-dormant pseudoexon.

The mutations that created the pseudoexons illustrated in Fig 2C were usually within the pseudoexon and affect auxiliarly splicing motifs but five mutations lie outside the pseudoexon and enhance the recognition of the polypyrimidine tract or branch point of the 3’pss and in addition some of the pseudoexons were activated by mutations of flanking 5’ or 3’ ss (Table S4, sheet 5, column I).

References

Dhir, A. and Buratti, E. (2010) Alternative splicing: role of pseudoexons in human disease and potential therapeutic strategies. FEBS J, 277, 841-855.

Vaz-Drago, R., Custodio, N. and Carmo-Fonseca, M. (2017) Deep intronic mutations and human disease. *Hum Genet*, **136**, 1093-1111.
