# Supplementary material for: Background splicing as a predictor of aberrant splicing in genetic disease
Source: RNA Biol. 2022 Feb 19;19(1):256–65. doi: 10.1080/15476286.2021.2024031 (PMC8865296; doi:10.1080/15476286.2021.2024031)
Supplement: Supplemental Material [file KRNB_A_2024031_SM7960.zip › Supplementary information/Appendix_6_unusual_aberrant_splicing_.docx]

**Appendix 6**.

Eight of the 298 splice site mutations chosen from DBASS (see main text) do not fit well into the formats of Figure 1A and Table S2 because the results are more complex. What most of these examples have in common (see below) is the activation of exon skipping that also involves the activation of css at a considerable distance from the ss mutation (which is unusal). Often the activated css are of the opposite splice to the mutated splice site ie a 3’css activated by a 5’ss mutation or a 5’css activated by a 3’ss mutation (this is also unusual). The css differ in strengh from weak to strong to exclusive use, as indicated. Overall our standard method of analysis (Fig 1) was good at predicting the exon skips. However, we needed to introduce a secondary analysis to identify the exon skips caused by css activation.

AGL

3’ssExon 34 weak 3’css

Exon 32 5’ss

Exon 33 5’ss

CDKN2A

3’ssExon 2 exclusive 3’css

β5’ss

Exon 1α 5’ss

COLQ

3’ssExon 17 weak 5’css

Exon 16 5’ss

Exon 15 5’ss

EVC

3’ssExon 4 exclusive 3’css

Exon 2 5’ss

Exon 3 5’ss

F8

3’ssExon 16 exclusive 3’css

Exon 14 5’ss

Exon 15 5’ss

INVS

Exon 12 5’ss

Exon 13 5’ss

3’ssExon 14 strong 3’css

TYMP

Exon 1 5’ss

3’ssExon 2

3’ssExon 3 exclusive 5’css

XPA

Exon 2 5’ss

Exon 3 5’ss

3’ssExon 5 css strengths not clear

3’ssExon 4

1

2

3

Exon 2 5’ss

Exon 3 5’ss

3’ssExon 5

3’ssExon 4

4

The secondary analysis suggested itself from the similar patterns seen for AGL, and INVS (and F8 to some extent). The primary analysis is as normal (Fig 1A), which led in all three cases to the identification of the 5’ss of the first exon (in these diagrams) as likely to be involved in aberrant exon skipping (see below). The secondary analysis looks for all of the partner ss of the 5’ss of the first exon in order to identify any other sites that might also participate in the exon skipping result. For XPA and TYMP, which have mutations of the 3’ss, we did a similar secondary analysis of the 3’ss of the last exon. The COLQ css were identified by the primary analysis only but we also did a secondary analysis as a control. There was no real agreement between the CDKN2A experimental results and the Snaptron data.

AGL

Table S2 DBASS3 index row 151

PMID: 10925384

The authors report that a mutation of the 5’ss of exon 33 caused it to be skipped by splicing between exons 32 and 34 plus they also reported a minor skipping event between exon 32 and a 3’css in exon 34.

3’ssExon 34

Exon 32 5’ss

Exon 33 5’ss

The mutated 5’ss (shown in red) CCTgt aa(g>a)a is 100382054 (hg 19) and its partner 3’ss of exon 34 is 100382153. We would normally look for the background 5’ss partners of 3’ss 100382153 in order to predict the likely effect of this mutation. These are shown below and as can be seen the single exon skip (in yellow) has the most reads of all of the background splice sites (290 reads, column O), so this part of the analysis agrees with the author’s results.


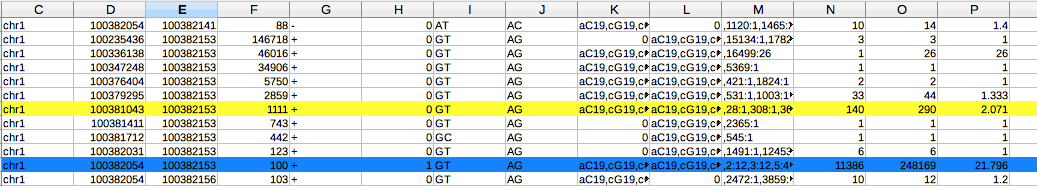


We usually look at the partners of a mutated splice site in case there is a strong ass that would also need to be analysed. The partners of the 5’ss of exon 33 (100382054) are listed below.


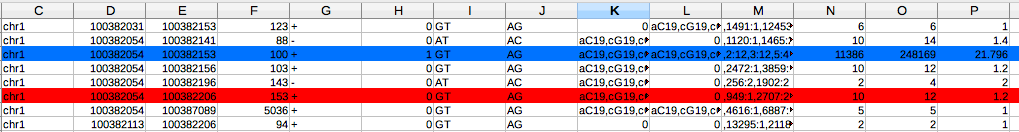


From above it can be seen that there are no ass but it can be seen that the 3’css identified by the authors (100382206, in red) is one of the bss for the 5’ss of exon 33 . This analysis indicates that the bss 100382206 would be a strong candidate for activation if the 3’ss of exon 34 was mutated but does not really explain why it is involved in the skipping of exon 33 as a result of a mutation of the 5’ss of exon 33 (see diagram). The rows in column G marked – are from the opposite strand to AGL and can be ignored.

As a secondary analysis we looked at background reads involving the 5’ss of exon 32


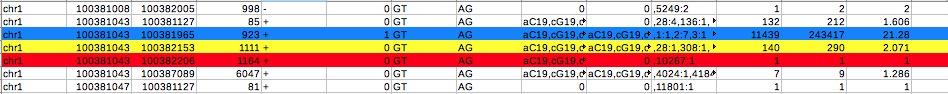


This analysis again shows that there are 290 reads between exon 32 and the 3’ss of exon 34 (as expected) and also shows 1 read between exon 32 and the 3’css discovered by the authors. Our working assumption is that this ratio of 290:1 will approximate to the ratio of skipping involving the 3’ss and 3’css of exon 34.

Consequently, Snaptron shows that the css identified by the authors by careful experimentation is an active bss that splices to the 5’ss of exon 33 (12 reads) and the 5’ss of exon 32 (1 read). This is hardly a strong prediction for exon skipping by this 3’css but is consistent with the finding that it was only detected as a relatively faint RT-PCR band compared to skipping via the 3’ss of exon 34.

CDKN2A

Table S2 DBASS3 index row 138.

PMID: 15856016

CDKN2A

3’ssExon 2 exclusive css

β5’ss

Exon 1α 5’ss

The authors report that a mutation of the 5’ss of exon 1β (21994137) causes the activation of a 5’css 12 bases before the start of exon 1β (21994341) which then splices to a 3’css in exon 2 (21970939). For this gene exons 1β and 1α both splice to exon 2 in a mutually exclusive fashion (explained by the authors). We therefore first looked for the 5’ss partners of the 3’ss of exon 2 (21971208), rather than the partners of the 3’ss of exon 1α. As expected there were most reads between exon 2 and the alternative 5’ss of exons 1β and 1α. There were also 63 further 5’bss partners for exon 2, with a range of reads from 1 to over 11,000. None of these 5’bss matched the 5’css reported by the authors (21994341). An unrestricted search for this 5’css showed that it matched a bss with single reads to 3 different 3’bss (but not to the 3’ss of exon 2 nor to the 3’css the authors discovered). The 3’css reported by the authors (21970939) matched a 3’bss within the Snaptron database with low reads to a small number of sites. Consequently, the Snaptron data does not predict the activation of these css by the indicated mutation (as far as we can see) but it does show that the css discovered by the authors have some background activity.

COLQ*-*19

Table S2 DBASS5 index rows 147 and 148

PMID: 10441569

3’ssExon 17

Exon 15 5’ss

Exon 16 5’ss

The authors report that a COLQ 5’ss mutation of exon 16 primarily causes a single exon 16 skip mediated by the upstream 5’ss of exon 15 but also mediated by two minor 5’css in exon 15 (5’CSS 15497424 and 15497435). This example although relatively unusual works OK with our normal approach (Fig 1A) which is to predict the likely effect of mutation of the 5’ss of exon 16 (hg19 15495335

TGGgt(a>g)g gg) by looking for 5’ bss partners of the 3’ss of exon 17 (15493221), as illustrated below. Please note that the 3’ss are in column D and the 5’ss in column E.


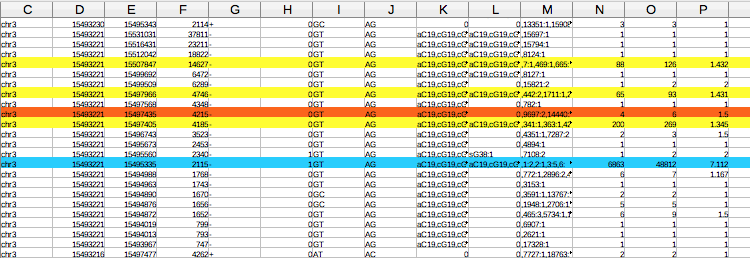


The Snaptron data shows that the background ss with the most reads is for

a single skip (269 reads, column O), which agrees with the experimental result. There are also 6 reads for one of the css that was reported (in red) and the other css has reads in the larger database SRAv2 (data not shown). Again, Snaptron shows that the css identified by the authors are active bss that were detected by careful experimentation but are not such an obvious prediction compared to the single exon skip, which is consistent with the experimental results.

As a control we also did a secondary analysis where we looked for the partner ss of the 5’ss of exon 1.


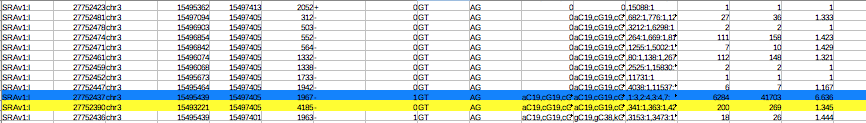


This showed 269 reads for skipping to the 3’ss of exon 17 but to no other site in this exon.

EVC

Table S2 DBASS3 index row 107

PMID: 26621368

3’ssExon 4

Exon 2

Exon 3 5’ss

The authors report that a mutation of the 5’ss of exon3 (hg19 5731118 CGGgtg a(g>c)a) inhibits splicing between exon 3 and the 3’ss of exon 4 (5733151), as expected. Remarkably the mutated 5’ss splices exclusively to a 3’css (5733220)in exon 4. Our normal approach, which is to look for alternative partners of the 3’ss of exon4 (5733151), indicates that the 5’ss mutation of exon 3 would cause it to be skipped as there are most reads (28) for this event in column O below.


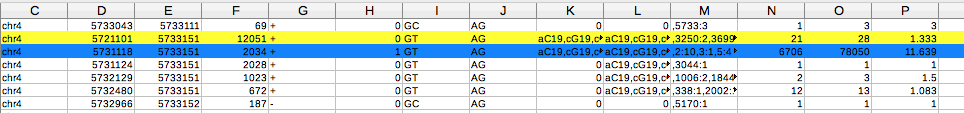


We also looked for all of the 3’ss partners of the 5’ss of exon 3 (see below)


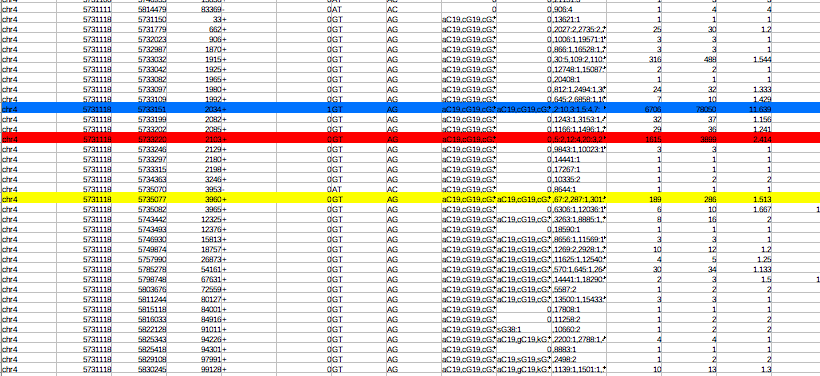


This reveals that the 3’css discovered by the authors is a very strong background partner to the 5’ss of exon 3 (shown in red, 3899 reads column O).

The authors’ results suggest that the +5 mutation inhibits splicing to the usual 3’ss partner of exon 4 but not to the alternative 3’ss within exon 4. The authors express some reservations about their results as they used a minigene construct because of the unavailability of patient RNA. Our method of analysis does not predict the authors results (it favours a single exon skip based on the assumption that the mutation would inhibit all splicing by the 5’ss of exon3) but the Snaptron database does show that the css identified by the authors is a highly active background ss, but gives no further insight into the unusual effect of the 5’ss mutation.

F8

Table S2 DBASS3 index row 160.

PMID: 11380640

3’ssExon 16

Exon 14 5’ss

Exon 15 5’ss

The authors report that a mutation of the 5’ss of exon 15 (154134694, ATG(g>t)tgagt) activates an upstream 5’ss but it doesn’t splice at all to the 3’ss of exon 16 (which would normally be expected) but to a 3’css (154133252).

We would normally identify the background 5’ss partners of the 3’ss of exon 16 (154133299) in order to predict the effect of the 5’ss mutation of exon 15. In this case there are only reads (14882)for splicing with the partner 5’ss 154134694 ie there are no background reads.


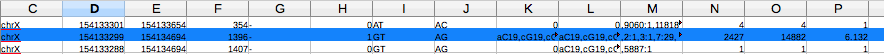


In the larger SRAv2 database there are 48K reads for normal splicing but again no reads for any variants. This is an unusual result because although there are quite a few examples of intron ss that have no background reads in Table S2, these are nearly always associated with relatively low intron reads. So Snaptron indicates that the 3’ss of exon 16 may have very high fidelity for splicing to the 5’ss of exon 15, which might help to explain the authors’ unusual results.

We also looked for the background partners of the exon 15 5’ss.


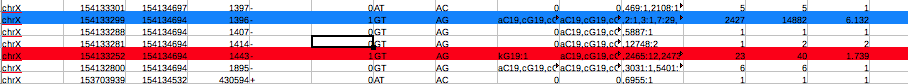


This identifies the css discovered by the authors as the next strongest partner for exon 15 5’ss with 40 reads after the normal 3’ss (with 14882 reads).

As a secondary analysis we looked for the partner ss of the 5’ss of exon 14. All of the sites were within the downstream intron, there were no background reads for downstream exon skipping (data not shown).

Overall, Snaptron gives some insight as to why the 3’ss of exon 16 is not used for aberrant splicing when its partner 5’ss is mutated (it seems to have very high fidelity for only its normal 5’ss partner) and also indicates that the 3’css of exon 16 might be used instead of the 3’ss of exon 16 because it is the next most active alternative 3’ss in this region. However, there are no supporting background reads to indicate that this 3’css is likely to splice with the 5’ss of exon 14

INVS

Table S2 DBASS index row 158.

PMID: 11935322

Exon 12 5’ss

Exon 13 5’ss

3’ssExon 14

The authors report that a mutation of the 5’ss of exon 13 activates splicing between the 5’ss of exon 12 and the 3’ss of exon 14 (this is a common effect of ss mutations). Unusually there is also splicing in roughly equal amounts (according to the RT-PCR band intensities) between the 5’ss of exon 12 and a 3’css within exon 14.

The mutated 5’ss (shown in red) ATGg(t >c)aggt is 103046886 (hg 19) and its partner 3’ss of exon 14 is 103054607. We would normally look for the background 5’ss partners of 3’ss 103054607 in order to predict the likely effect of this mutation.


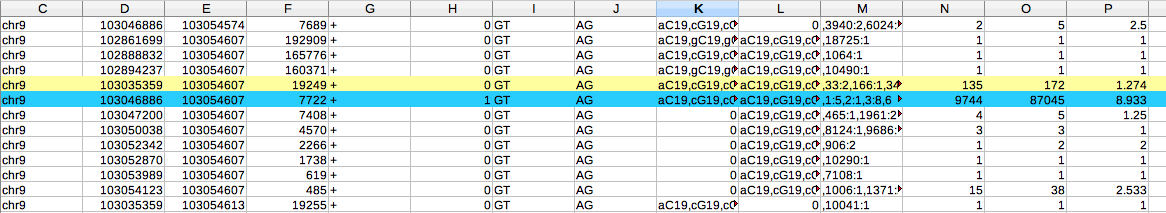


The Snaptron data has most background reads (172, column O) for the exon skip, in agreement with the author’s results.

We also looked for the partners of the 5’ss of exon 13 in case there were any strong alternative ss.


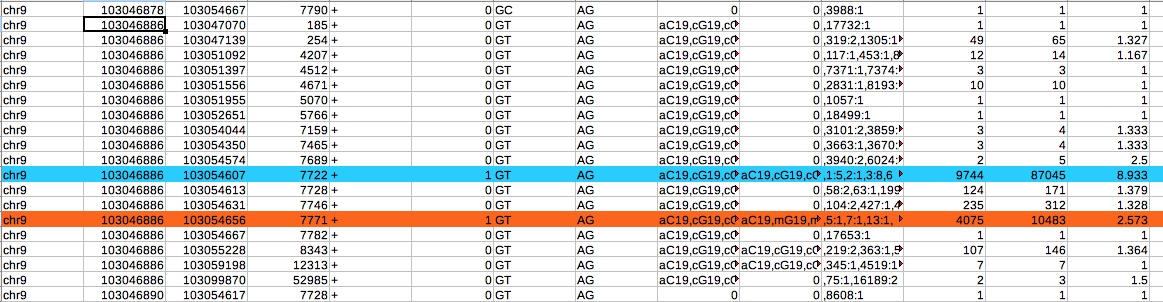


This screen shows that the 3’ss 103054656 (which is the 3’css identified by the authors) has 10483 reads with the 5’ss of exon 13 , which is over 10% of the intron reads of 87045. Consequently, the 5’ss mutation of exon 13 is also likely to cause this minor 3’ass to splice with alternative 5’ss. Analysis of this 3’ass shows that its next most common partner is the upstream 5’ss of exon 1 (shown


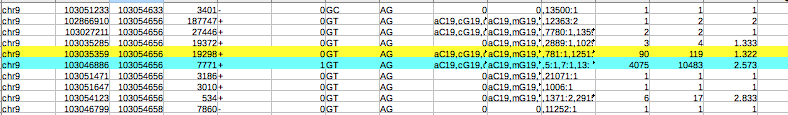


in yellow) with 119 reads.

As a secondary screen we also looked for the partners of the 5’ss of exon 12.
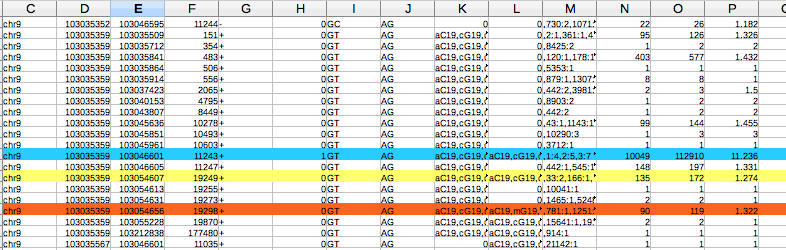


This gives a better overall picture that confirms there are 172 reads for the exon skip to the 3’ss of exon14 and 119 reads to the 3’css discovered by the authors. Our assumption is that these would be used roughly in the ratio of 172:119, which accords with the experimental results.

TYMP

Table S2 DBASS5 index row 437

PMID: 14757860

Exon 1 5’ss

3’ssExon 2

3’ssExon 3

The authors report that a mutation of the 3’ss of exon 2 (50967768acc a(g>c)GGG) causes exon skipping by activating splicing between the 3’ss of exon 3 (50967040) with a 5’css within exon 1 (50967955).

We would normally look for the background partners of the 5’ss of exon 1 (50967924) in order to predict the likely effect of this mutation, which would be the activation of both single (336 reads) and double (1140 reads) downstream exon skipping (see below).


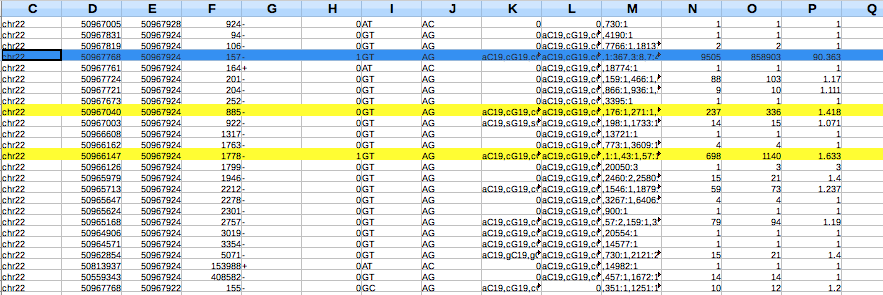


We would also check the 5’ ss partners of the 3’ ss of exon 2 in case of complications with alternative splicing (see below).


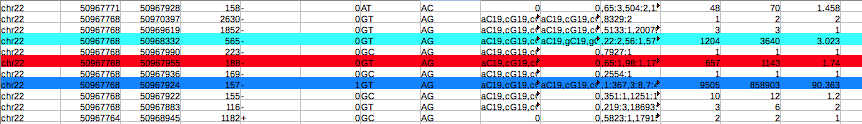


This analysis shows that the 5’css disovered by the authors (marked in red) is a strong bss for the 3’ss of exon 2, as is another site marked in light blue. Both of these sites would be good candidates for 5’css activated by mutations of the 5’ss of exon 1.

As a seondary analysis we looked for the partner ss of the 3’ss of exon 3. There are 130 reads between the 5’css of exon 1 with exon 3, which compares quite well to the 336 background reads between the 5’ss of exon 1 with exon 3.


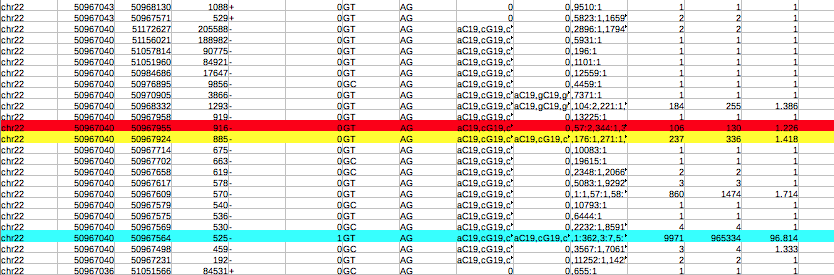


Our normal assumption would be skipping between the 5’css or the 5’ss of exon 1 to exon 3 to be roughly in the ratio of 130:336 ie we have no explanation for the experimental finding for the exclusive use of the 5’css

So the Snaptron data shows that the css detected by the authors is a relatively active bss that splices to the 3’ss of both exons 2 and 3. It also shows that the 5’css of exon 1 has a surprisingly large amount of background reads for exon skipping to exon 3 compared to the 5’ss of exon 1. However, the Snaptron data gives no further insight to the authors observation that the 5’css of exon 1 was exclusively preferred to the 5’ss of exon 1. Overall Snaptron most strongly predicted a double exon skip, it is not clear if this was tested.

XPA

Table S2 DBASS5 index row 640.

PMID: 8595429

Exon 2 5’ss

Exon 3 5’ss

3’ssExon 5

3’ssExon 4

3

2

1

Exon 2 5’ss

Exon 3 5’ss

3’ssExon 5

3’ssExon 4

4

The authors identify four aberrant transcripts caused by the exon 4 3’ss mutation (100449544, tttca(g >t)AGA). Transcripts 1, 2, and 3 were sequenced and show to have spliced between the normal 3’ss of exon 5 and a 5’css in exon 2, a 5’css in the intron just after exon 2 and the normal 5’ss of exon 3, as illustrated above. Transcript 4 shows splicing between the normal 5’ss of exon 2 and a 3’css in exon 4. This is shown separately for reasons of clarity. The hg19 reference numbers of the relevant ss of exons 2 to 5 are:

3’ss exon 5’ss

- 2 100455930

100451922 3 100451815

100449544 4 100449377

100447323 5 -

We would normally look for the background partners of the 5’ss of exon 3 to predict the effect of a mutation of the 3’ss of exon 4. This analysis shows that the splicing events with the most reads after the 3’ss of exon 4 (shown in blue) are a 3’bss at position 100449542 with 61 reads, a single exon skip to exon 5 with 126 reads and a double exon skip (to exon 6) with 26 reads.


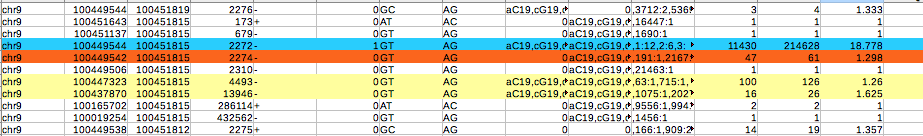


The exon skip with 126 reads is the same as transcript 3 discovered by the authors and the 3’bss 100449542 is the 3’css that the authors also discovered (transcript 4) but they found it spliced to exon 2 rather than exon 3.

We also analysed the partners of the 3’ss of exon 5 as a secondary analysis.


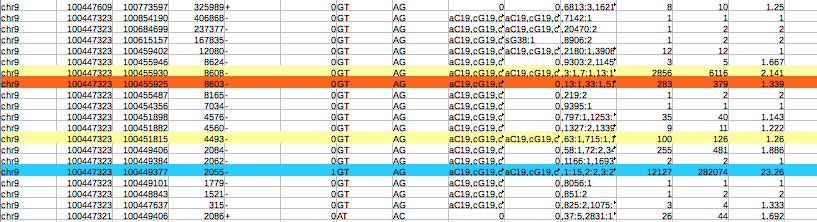


This screen shows that the 3’ss of exon 5 normally has 3668 reads to the 5’ss of exon 3 and 6116 reads for the double skip to exon 2. The line red shows that there are 379 reads for transcript 2, which is also likely to be increased in usage as a result of the mutation of the 3’ss of exon 4 blocking a competing splicing events. We didn’t find any background reads for transcript 1.

So overall Snaptron would predict that the mutation the authors investigated would promote single and double exon skipping with some minor activation of two of the three css that the authors discovered. The authors did not discuss their unusual observation of exon skipping via css rather than intronic ss. The reads for the single and double exon skips are quite high and so might also be found in control samples. Perhaps for this reason they were not mentioned.
