# Supplementary material for: Background splicing as a predictor of aberrant splicing in genetic disease
Source: RNA Biol. 2022 Feb 19;19(1):256–65. doi: 10.1080/15476286.2021.2024031 (PMC8865296; doi:10.1080/15476286.2021.2024031)
Supplement: Supplemental Material [file KRNB_A_2024031_SM7960.zip › Supplementary information/supplementary figure and table legends.docx]

**Description of supplementary figures, tables and appendices**

**Supplementary files Description**

Fig S1 BRCA1 Background splicing data used to make Table 1 and illustrated in Fig 1B,C

Table S1 Discusses discrepancies between BRCA1 experimental and background splicing data as indicated in Table 1

Table S2 css Individual rows of each Table compare background splicing data with css identified by experiment in DBASS, BRCA2 and DMD. This data is summarised in Table 2 and part of Table 3.

Table S3 exon skips Each row reports an exon skip analysis that are summarised in Table 3

Tables S4 de novo ss, pseudoexons Data used to make Fig 2 columns 1-8 and further

and recursive ss discussed in Appendices 2 and 3

Table S5 cancer Data used to make column 9 of Fig 2 and further discussed in Appendix 4

Table S6 GTEx Analysis that shows that bss are equally used in the GTEx RNA database of normal tissue (see Discussion)

Table S7 DMD Background splicing comparison with DMD exon skipping by antisense oligonucleotides (Discussion and Appendix 5)

Appendix 1 Protocol for using background splicing data to predict the effect of intron splice site mutations

Appendix 2 Summarises and analyses the de novo mutations and pseudoexons listed in the DBASS database.

Appendix 3 Further discusses the match between bss and recursive ss

Appendix 4 Further discusses the match between aberrant splicing events in cancer with bss

Appendix 5 Further discusses the contribution background splicing data might make to splicing therapy

Appendix 6 Discusses 8 out of 298 intron ss mutations from DBASS that have more complicated effects than those illustrated in Fig 1A.

**Supplementary Figure and Table legends**

**Figure S1** was made by downloading the RNA splicing information for BRCA1 using the link <http://snaptron.cs.jhu.edu/srav1/snaptron?regions=brca1> , as described in Appendix 1. Many of the downloaded columns were deleted to leave those showing the chromosome number (column A), the original columns headed start and end were swapped in order to show the 5’ss on the left (column B) and 3’ ss on the right (column C) the distance between the splice sites (column D) and column E shows the strand is negative. In some rows column E is +, these rows should be ignored as they are transcripts from the wrong strand. Column F was inserted by us and column G shows the coverage sum (changed to reads) from Snaptron for the splicing events on that row. The rows were edited down from over 6000 entries to those splicing events involving the major 3’ss (sheet 1) or 5’ss (sheet 2) of BRCA1 (see Appendix 1). The ss were ordered high to low so that ss at the 5’end of BRCA1 are listed first. The splicing events with the most reads in column G are shaded blue and these show intron removal, major alternative splicing events are shaded light blue. In sheets 1 and 2 the 5’ intronic ss or the 3’ intronic ss (sheet 2) is shaded red if there have been experimental reports of its mutation. Details of the experiment and the sequence of the 5’ and 3’ ss are shown in column H and I. cDNA numbering is shown in column K. Red shading in column G identifies the reads for bss of normal BRCA! that match the experimental results given in column H. Yellow shading shows background skipping events between an intron 3’ss and upstream intronic 5’ss (sheet 1) or between an intron 5’ss and downstream 3’ss (sheet 2).

**Table S1** discusses the results from Table 1 where the experimental and background ss data match poorly or less well (shaded). It also contains data from Fig S1 to illustrate the discrepancies.

**Table S2** DBASS index

Columns A to E are downloads from DBASS5 (sheet 1) and DBASS3 (sheet 2) following the command view all (http://www.dbass.org.uk/). Column F describes whether the mutation activates a css, a pseudoexon or creates a de novo splice site. Column G - occasional notes. Colours (column A) show the DBASS entries chosen for comparison with Snaptron RNA seq data. For css (yellow), only the first pathogenic mutation for each syndrome was chosen for analysis although all aberrant splicing events caused by the same mutation were included. All unusual css (green) and pseudoexons (blue) examples were analysed and the first 40 different examples of de novo splice sites caused by enhancing mutations (orange). Please note that the order shown here is not necessarily the same order seen by a search for individual genes. Unusual css refers to those generated by mutations outside the 5’ or 3’ consensus ss and unusual pseudoexon refers to pseudoexons generated by mutations that are not de novo ss mutations. An enhanced de novo ss refers to a mutation that enhances already existing 5’GT, GC or 3’AG ss, whereas a created de novo ss refers to a mutation that creates 5’ GT, GC or 3’AG ss. A summary of the numbers of each type of splicing mutation in DBASS is given at the bottom of each sheet.

**Table S2** 5’css and 3’css (Summarised in Table 2)

Column A. Complete list of medical syndromes in DBASS5 and DBASS3 caused by css activation as a result of mutations of any of the bases of the 5’ss consensus sequence CAG/guragu (sheet 1) or of the 3'ss consensus sequence yyyaag/G (sheet 2). Column B lists the mutated gene and column C gives the DBASS link (may need to be copied and pasted) to the experimental report and sequence data for the splice site mutation and the activated css. Columns D to H give the chromosome and genomic locations of the canonical 5’ and 3’ss, details of the mutation and the genomic location of the activated css (hg19). Column I shows the distance of the css from the mutated intron ss, where - is upstream and unmarked is downstream. Column J shows whether the reported css in column I is listed in Snaptron as a background splice site. Column K (read rank) describes how well the experimental data matches the RNA sequencing reads. For example, 1(4) means there are four background ss within 1000 bases of the mutated canonical ss and the css matches the background ss with the greatest number of reads. Column L (distance rank) describes how near the css is to the mutated intron ss. For example 2(6) means that there are 6 background ss within 1000 bases of the mutated canonical ss and that the bss that matches the css is second nearest. Column M (notes) provides additional information. Column N shows whether the ss mutation also caused exon skipping, as indicated by Yes or No or nr (not reported/tested). Columns O (normal reads) shows the number of sequencing reads between the intron 5' and 3'ss and column P (css reads) shows the number of reads between the bss that matches the css and its intron ss partner. If the experimental css did not match a background ss then the largest read number for a bss within 1000 bases of the mutated intron ss is shown in brackets. Column Q shows the number of background sequencing reads for single exon skipping of the mutated exon. Shading highlights cases where the exon skipping reads are greater than the css reads. Different coloured shading is reflective of column N. Column R list snaptron reads for multiple exon skipping, column S identifies introns where skipping could not occur, column T identifies bss within 1000 bases of the mutated intron ss that have reads that are greater than the bss that matches the reported css. At the bottom of each sheet there is an analysis that includes a discussion of all examples where the experimentally reported css did not match a background ss. The method used to determine the probability values for the match between css and bss with top reads is also explained.

**Table S2** DBASS5w and DBASS3w (Summarised in Table 2)

Medical syndromes (column A) caused by relatively weak mutations outside the core conserved bases of normal 5'ss and 3’ss that nevertheless result in css activation. Column B lists the mutated gene and column C gives the DBASS link, which references the original paper and gives sequence data about the splice site mutation and the activated css. Columns D to H give the chromosome and genomic locations of the relevant 5'ss, 3’ss and css and the position of the splicing mutation relative to the mutated ss. Column I shows the distance of the css from the normal ss, where - is upstream. Column J shows whether the reported css is listed in Snaptron as a background splice site. Column K gives the read rank where 1(14) means that there are 14 background ss within 1000 bases of the mutated intron ss and that the background ss that matches the css has the highest number of reads. Column L gives the distance rank where 3(14) means that the background ss that matches the css is the third nearest of 14 background ss to the mutated ss. Column M shows whether the ss mutation also caused exon skipping, as indicated by Yes or No or nr (not reported/tested). N and O show the number of sequencing reads for normal splicing between the 5' and 3'ss and for splicing with the css. Column P shows the number of reads for the bss with the highest reads, other than the bss that matches the css (these reads are shown in column O). Columns Q and R list the background sequencing reads for single and double exon skipping. Column S has occasional notes. At the bottom of each table we discuss css that do not match bss. For those css that do match we calculate that css activated by weak splice site mutations on average match bss with relatively more reads than the css activated by strong ss mutations (Table S2 5’css and 3’css).

**Table S2** BRCA2 (Summarised in Table 2)

Comparison of the experimental effect of splice site mutations of BRCA2 with Snaptron RNA seq data. Column A lists the normal 5’ or 3’ splice sites (hg19) that have been impaired by mutation (the exact mutation may differ between publications). The second column B (experimental summary) indicates whether the mutation caused css activation, exon skipping or both. Column C indicates whether the experimentally identified css (from column 2) is present in the Snaptron RNA splicing data as a background splice site. Column D shows the css rank and for example 7(10) for row 5 of this column means that Snaptron identified ten bss within 1000 bp (upstream or downstream) of the 5’ss 32889805 and that the bss that matched the experimentally identified css had the seventh most reads. Column E shows the reads of those bss that match the css. Column F lists the reads of the highest scoring background splice site within 1000 bases of the mutated splice site. Columns G and H list background reads for single and double exon skipping. The shaded boxes indicate the RNA splicing reads that are not a good fit to the experimental results in column B. Some of these results are discussed in column K and at the bottom of the Table.

**Table S2** DMD (Summarised in Table 2)

Comparison of the experimental effect of splice site mutations of DMD with Snaptron RNA seq data. Columns A to K are similar to those already explained in Table S2 BRCA2. Columns M to Q are a repeat analysis using the larger database SRAv2.

**Table S3**

**Index**. Column A lists, by gene order, reports of exon skipping in response to ss mutations. (from Kralovicova & Vorechovsky 2007 PMID: 17881373). Column B shows whether each paper reports a 5’ or 3’ or both a 5’ and 3’ skipping event. For the exon skipping events caused by 5'ss mutations we chose a single example from every gene (blue shading, first on the list if more than one example) to compare with the Snaptron database (Table S3, sheet 2, 5’skip). We could not analyse some of the examples for the reasons given in column C and we did not use any of the examples that reference Krawczak et al 2007 (human gene database). Because there were fewer examples of exon skipping caused by 3'ss mutations we analysed all of the examples listed here (orange shading) and also used those references from the human gene mutation database that are indicated by Krawczak et al 2007. We also included all of the 3'ss skipping mutations from Table S1 of Divina et al 2009 PMID: 19142208 (indicated in Table S3, sheet 3, 3’skips).

**5’skips**. These examples were systematically chosen from the exon skip database made by Kralovicova & Vorechovsky 2007 PMID: 17881373 (see Table S3 Index). Columns A and B describe the genes and give references. Columns C to G show the chromosome, position of the two nearest upstream intron 5'ss (D) the mutated intron 5'ss (E), the mutation change (F) and the 3'ss intron position (G) (hg19). Column H gives the reads for normal splicing between the 5' and 3'ss (E to F). Column I lists single skip reads (D to G) between the 3'ss and the first intron 5'ss that is upstream to the normal intron 5’ss partner in column E. Column J double skip reads between the 3'ss and second upstream intron 5'ss. Column K lists the highest reads between the 3'ss and a background 5'ss within 1000 bases of the 5'ss. Column L highest reads between the 3'ss and a background ss more than 1000 bases from the 5'ss. Column M lists whether a css was also reported and column N whether the css matches a background ss. Column O summarises the experimental results and comments on how these match the RNA sequencing data and see discussion at bottom of the Table.

**3’skips**. These examples were from Kralovicova & Vorechovsky 2007 PMID: 17881373 and a further 7 (marked in orange) from Divina et al 2009 (10). Columns A and B describe the gene and give a reference. Columns C to G show the chromosome, position of the 5'ss, the mutated 3'ss, the mutation change and the positions of two intron 3'ss that are downstream (hg19). Column H gives the reads for normal splicing between the 5' and 3'ss (D to E). Column I single skip reads between the 5'ss and the immediate downstream 3'ss. Column J double skip reads between the 5'ss and second downstream 3'ss. Column K highest reads between the 5'ss and a background 3'ss within 1000b of the 3'ss. Column L highest reads between the 5'ss and a background ss more than 1000b from the 3'ss. Column M lists whether a css was also reported and column N whether the css matches a background ss. Column O – experimental results and notes on their match with the RNA sequencing data and see discussion at bottom of the Table.

**Table S4** de novo mutations, pseudoexons and recursive ss (Summarised in Appendix 2)

Sheets 1 (5’dn) and 2 (3’dn). These examples were taken from DBASS (Table S2 index) and were chosen for the reasons given at the bottom of each table. Column A: List of medical syndromes in DBASS5 and DBASS3 caused by de novo ss creation or enhancement. Column B lists the mutated gene and column C gives the DBASS link to the experimental paper and sequence data for the de novo ss mutation. Columns D to H give the chromosome and genomic locations of the intron 5’ and 3’ss and details of the mutation and the genomic location of the de novo ss (hg19). Column I shows the distance of the de novo ss from the canonical ss, where - is upstream. Column J shows whether the de novo ss was created or enhanced, where enhanced means that there was a pre-existing /gt, /gc (sheet 1) or ag/ dinucleotide (sheet 2), where / is the splice site (and see column G). Column K gives the read number for splicing between the intron 5’ and 3’ ss and column L gives the reads for splicing between the de novo ss and its intron ss partner. Column M and N give the read and nearness ranks for the de novo ss. Column O gives the largest number of reads for any splicing event involving the de novo ss (unrestricted).

Sheets 3 to 5. Pseudoexons as illustrated in Fig 2 A to C. For sheets 3 and 4 the column headings are similar to sheets 1 and 2 but also include details of the pseudosplice site partner. Sheet 5 describes the pseudoexon splice sites that are activated by mutations that do not generate de novo splice sites but instead often affect auxiliary splice sites.

Sheet 6 (recursive ss). Columns A to D are from Kelly et al 2015 PMID: 25897131 and show the first 20 or so entries of 2,389 unique recursive splicing sites identified in HUVECs. The base indicated (by chromosomal and strand location (hg19) corresponds to the +2 position after the RS junction. Columns F to M are from Snaptron and give the intron size (column F) the 5' and 3' ss coordinates of the intron (columns G,H), the intron splicing reads (column I) the recursive splice site coordinate (column J) and the reads between the recursive splice site and the partner intron splice site (column K). Column L shows the total number of background ss within the intron and the relative read number of the recursive splice site where 1 is top.

Sheet 7 (recursive ss). Columns A to F are downloaded from Fig S4 from Gazzoli et al 2016 PMID: 26670121. Column G shows the Snaptron reads for the recursive ss (identified by Gazzoli et al 2016) with the intron ss partner and column H gives the reads for the intron. Column I lists the number of background ss for each intron (number in brackets) and the read rank of the background ss that matches the recursive ss where 1 is top. Sheet 3 is a summary table (see Appendix 3)

**Table S5a** Cancer (Summarised in Appendix 4)

Worksheet 1 is a summary table discussed in Appendix 4. Worksheet 2 SF3B1 3’css Darman et al 2015. Columns A to G are taken from supplementary Table S2 of Darman et al., 2015 PMID: 26565915 which lists 895 aberrant splicing events resulting from mutations of spliceosome component SF3B1 in a range of cancers. For worksheet 1, 26 examples were filtered for 3'ass (css) activation (column D) and then filtered for those with highest ratio of aberrant to canonical sequencing reads (column F) and junction novelty (column G). Columns H to M summarise an analysis of these same aberrant splicing sites by using the Snaptron database. Column H gives the read rank, for example in row 3 the value 1/26 means that the 3'css (34144725 from column C) has the highest number of reads out of a total of twenty six 3'bss in Snaptron that splice with the intron 5'ss 34144042 (column B). Column I lists the nearness rank. For example in row 3 the value 1/26 means that the css 34144725 is the nearest of the 26 3'bss to the intron 3'ss 34144743. Column J is similar to column I but only considers intronic bss (not those that are downstream of the canonical 3'ss). Column K gives the Snaptron reads for intron splicing and column L gives the reads for aberrant splicing between the 3’css match and intron 5’ss, column M gives the ratio of K/L. Columns N to Q are from Darman et al., 2015 and show in which cancer(s) the aberrant splicing was detected. Conclusions are drawn at the bottom of worksheet 2 and see Appendix 4. Worksheets 3-5 show a similar analysis from Darman et al 2015 of 5’css activation, exon skipping and exon inclusion. Worksheets 6 to 8 are from DeBoever et al 2015. Worksheet 6 shows the first ten examples from Table S3 (Deboever et al. 2015) which lists 619 cryptic 3’ss located 10–30 bp upstream of canonical 3’ss from joint BRCA, CLL, and UM cancer samples with SF3B1 mutations. Worksheet 7 shows the first ten examples from the S4 File of DeBoever et al 2015, which lists 417 distal cryptic 3’SSs used more often in SF3B1 mutants from joint BRCA, CLL,and UM analysis. Worksheet 8 is a comparison of the css from Fig 4b of DeBoever et al 2015 with the Snaptron database. Worksheet 9 is from Table S17 of Suzuki et al 2019 who identified 1310 5’css that are activated by mutations of the U1 snRNA in SHH medulloblastomas. We have compared the first twenty or so of these plus the genes PTCH1, GLI2, CCND2 and PAX5, which are implicated in this cancer with the Snaptron database. Worksheet 10 gives the sequencing reads and ranking for the exon inclusions in EZH2 and BRD9 (see text) from Snaptron databases SRAv1 and GTEx.

Table S5b

Similar analysis to Table S5a except using the GTEx database from Snaptron.

Table S6.

Sheet 1. Columns A to D summarise how many background ss from SRAv1 (B), SRAv2(C) and Gtex (D) match the 35 cryptic splice sites of BRCA1 and BRCA2 combined (Tables 1 and S2). Columns F and G list the css and their positions for BRCA1 and columns O & P are for BRCA2. Columns H-J and Q to R show whether each of the css of BRCA1 or BRCA2 match bss from SRAv1, SRAv2 or Gtex. Columns K -M and T-V are from sheets 2 and 3 and show the ratio of the reads for intron removal divided by the css reads. Sheets 2 and 3 show details for the BRCA1 and BRCA2 analyses that are summarised in Sheet 1.

Table S7 (see Appendix 5).

Background skip reads for DMD exons. Each DMD exon is listed in column A and the coordinates of the 3’ and 5’ ss at the start and end of the exons (hg19) are listed in columns B & C. The size of each exon is shown in column D. Column E shows the classification of Wilton et al 2007 where 1 is an exon that was found experimentally to be easiest to skip with an ASO and 4 is an exon that was most difficult to skip. Column F shows our theoretical classification of how easy it might be to skip exons based on their background splicing data shown in columns H to J. Column I gives the background reads for the single skipping of each exon by normal DMD transcripts. Column H lists the number of reads for upstream multi-skipping events involving each exon when these reads are more than 50% of the single exon skip reads. Column K shows the downstream skipping events. For example for exon 3 (row 4) there are 22 reads for skipping exon 3 only, 39 reads for skipping exons 3 and 4 (as shown by 2nd skip comment in column J) and no reads greater than 11 for skipping exon 3 with any upstream 5’ss (column H). Exons classified as 1 (column F), might in theory be the easiest to skip because the background reads for single exon skipping are greater than the reads for alternative skipping events involving the same exon both upstream and downstream. Exons classified as 3 (column F) might in theory be the hardest to skip because there are reads for alternative events both upstream and downstream that are greater than the background reads for single exon skipping. Exons classified as 2 in general have reads for single exon skipping that are greater than the reads for either upstream or for downstream alternative events, but not both. The asterisks in column I show whether mutations of the 3’ss or 5’ss of each exon have been reported. For example for exon 2 row 3 the asterisk before 106 (number of reads for single exon skipping) indicates a report for a mutation of the 3’ss at the start of the exon. The effect of 3’ss mutations is shown in column K and the effect of 5’ss mutations in column G (further details in Table S2 DMD). Columns L to P are a repeat analysis using the larger SRAv2 database of spliced RNA. Column Q reports multiple exon skips determined by experiment in response to ASOs.
