# Supplementary material for: Background splicing as a predictor of aberrant splicing in genetic disease
Source: RNA Biol. 2022 Feb 19;19(1):256–65. doi: 10.1080/15476286.2021.2024031 (PMC8865296; doi:10.1080/15476286.2021.2024031)

**Table S1. Shaded examples from Table 1.**

**Table 1, row 5.**

Chen et al 2006 PMID: 16619214; Thomassen et al., (2012) PMID: 21769658 and Colombo et al., (2013) PMID: 23451180 report that a mutation of the 5’ss 41256138 causes the activation of a 5’css 41256200(hg19) at -62 , this deletes 62bp from the end of exon 7.


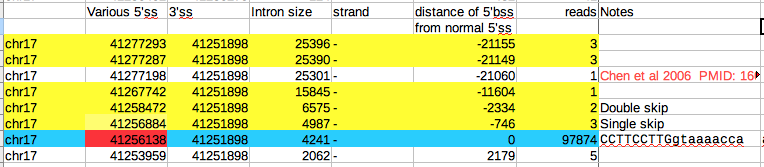


Above is from Fig S1 and shows all splicing events involving the the partner 3’ss 41251898 of the mutated normal 5’ss 41256138. This 5’ss also splices to an alternative 3’ss at 41251895, similar results to above. There are no background reads for the css at -62 in Snaptron SRAv1 although there is a single read for this event in the larger SRAv2 databse.

The main contradiction between experiment and background splicing in both SRAv1 and SRAv2 is that there are more background reads for single (3 reads) and double exon skips (2 reads) than for the experimentally identified css at -62. The RT-PCR primers listed in Table S2 of Thomassen et al (2012) anneal to exon 5 and 11 so they would have seen the single and double skips of exon 7 and of exons 6 & 7 predicted by Snaptron if they had occurred. Similarly Colombo et al 2013 used primers located in exons 5 and 8. There is a report of both exon 7 skipping and activation of css-62 by this 5’ss mutation(Steffensen et al 2014 PMID: 24667779), using minigene analysis. Overall the background splicing data underestimates the use of css -62 following mutation of the nearby 5’ss.

**Table 1, row 9, bss (41242251) with 115 reads not detected by experiment.**


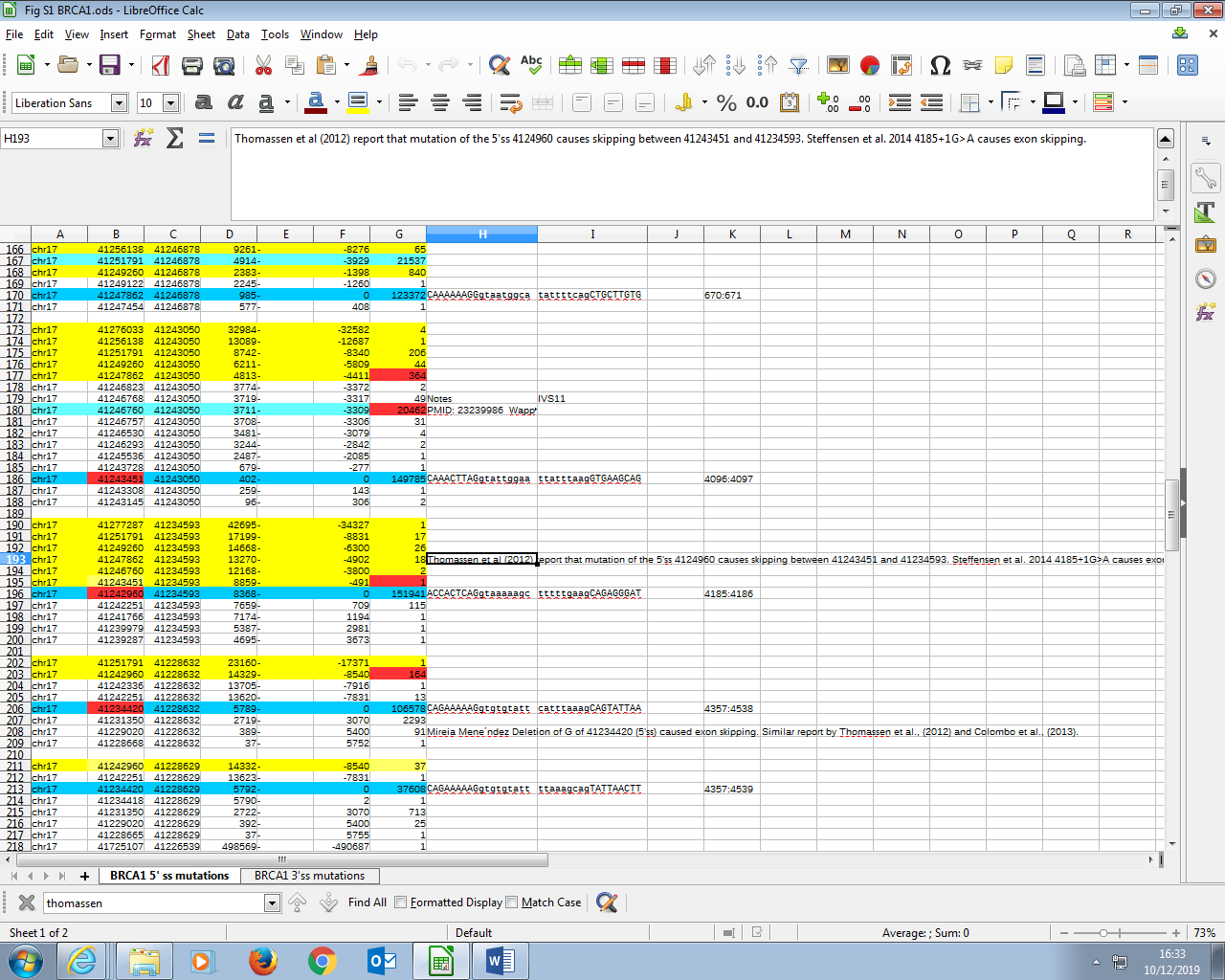


Thomassen et al., (2012) detected a skip as a result of a mutation of the 5’ss 41242960 and would likely have detected the css at 41242251 if it had been used at a reasonable frequency.

Consequently, the background ss at +709 is probably a false positive, with regards to css potential. The intron is quite large (8368 bases) and so is likely to contain recursive splice sites (see text).

**Table1 row 15**

**
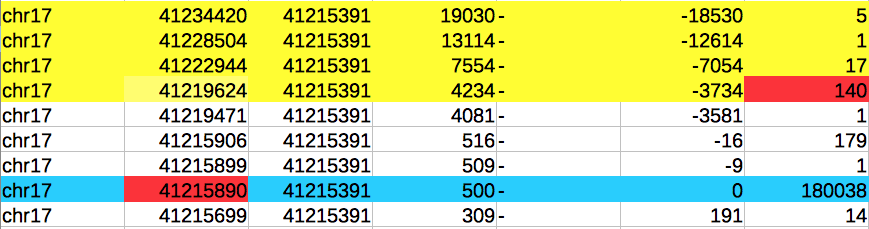
**

PMID: 23239986 Wappenschmidt et al (2012) report skipping (41219624 to 41215391) following mutation of the 5’ss 41215890+1G to C. Baert et al., 2018 PMID: 29280214 also report similar results. No groups have reported the activation of a bss at -16 (179 reads), indicating that it is a false positive.

**Table 1 row 16, bss (41215906) and double exon skip not detected by expt.**


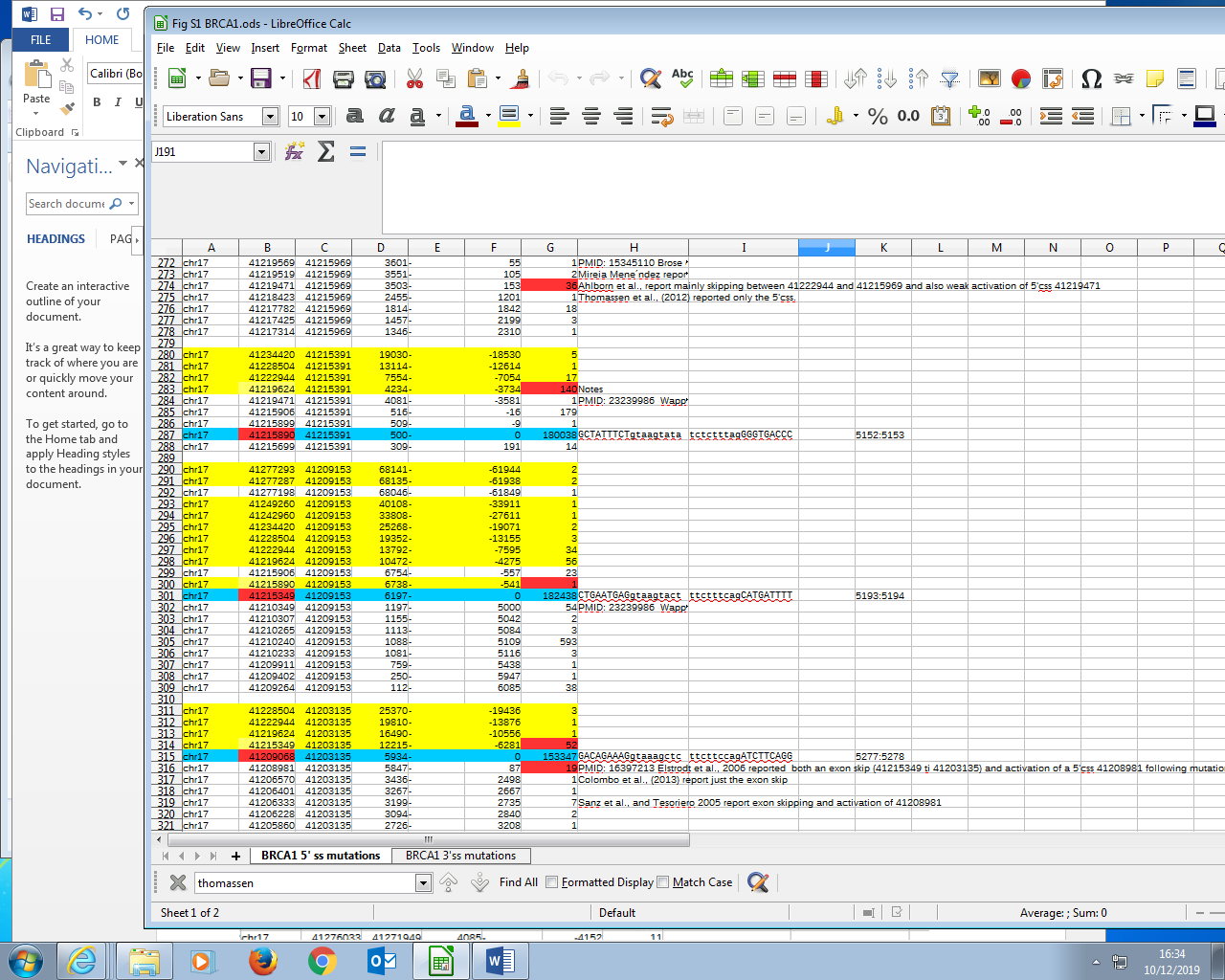


Wappenschmidt et al (2012) report that ivs19+2T>G causes exon skipping (Snaptron has 1 read for this, see above) but did not detect bss 41215906, which has 23 reads. Use of this predicted css would also cause exon skipping plus the additional deletion of the last 16 bases of the upstream exon. The primer used to detect the skip would also have detected the predicted css 41215906, if it had been used, so a possible false positive. Also Snaptron has 56 reads for the double exon skip between 41219624 and 41209153. The oligo listed as being used for the RT-PCR would not have detected this event (if it occurred).

Table 1 row 20.


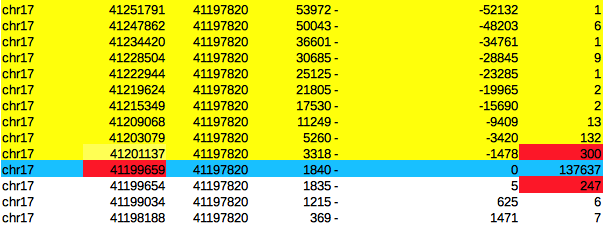


Four groups report that mutations affecting the 5’ss 41199659 caused exon skipping but only Yang et al 2003 detected activation of a css at +5, although they had a stronger RT-PCR band for the exon skip. The snaptron reads of 247 for the +5css and 300 for the exon skip predicts similar activation of both splicing events. Whiley et al 2011 studied a +5G>C mutation that would have destroyed the +5 css. Ladopolou et al 2002 studied the same G>A mutation subsequently analysed bt Yang et al 2003 and Rouleau et al 2010 studied a mutation 14 bases upstream from the end of exon 23. Yang et al 2003 analysed a homozygous mutation and G>A mutation at the end of the exon and found that the relatively weak mutation did not entirely prevent normal intron removal. They were not able to resolve normal splicing from use of the +5css by RT-PCR but did so by sequencing of isolated clones made from the RT-PCR band. Both Ladopolou et al 2002 and Rouleau et al 2010 had the added complication of studying a heterozygous mutation from patient material, which would have added to the difficulties (see above) of detecting activation of the +5 css.

**Table 1 row 21. Single exon skip not detected despite having far more reads (2622) than the detected css (5 reads)**


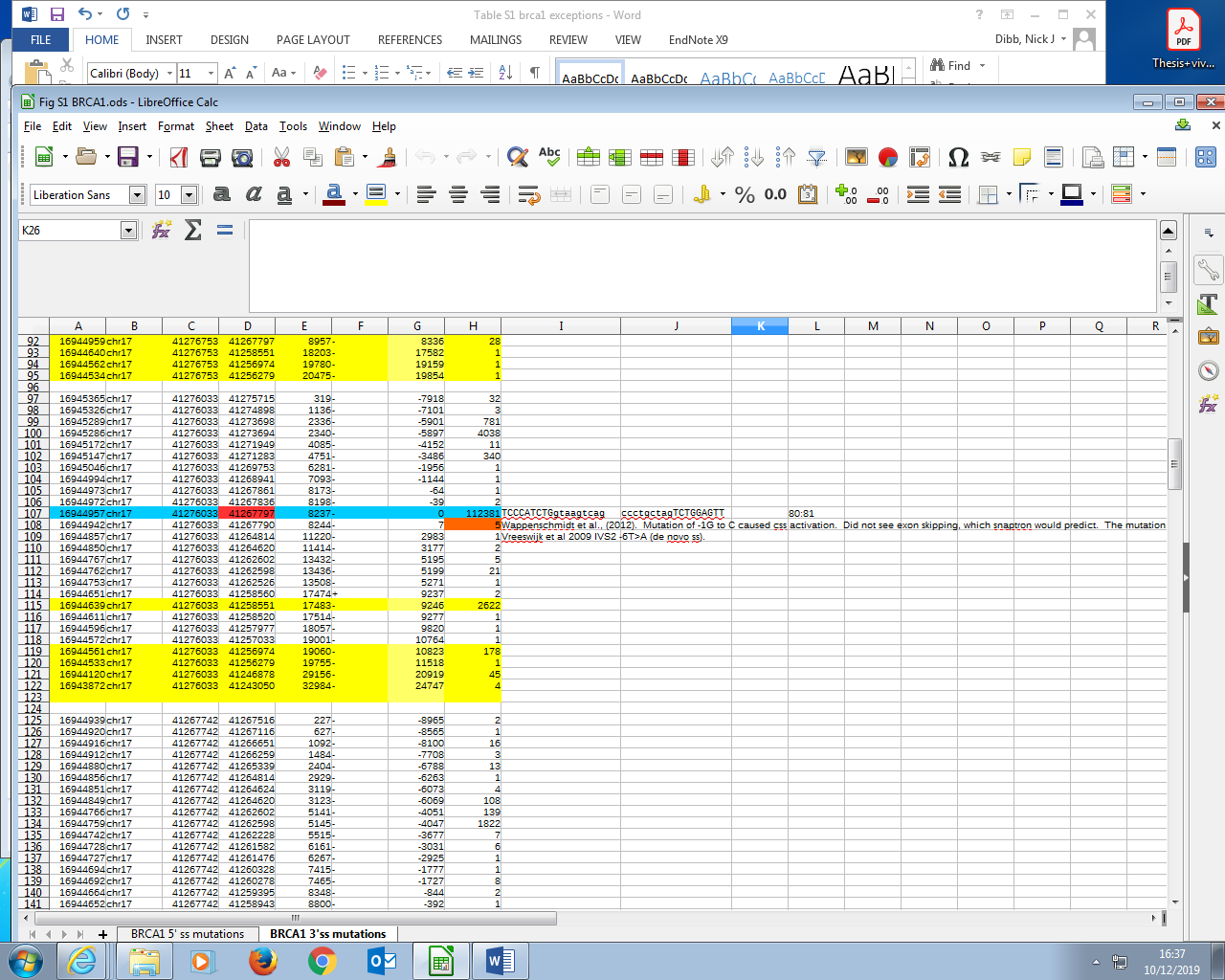


Wappenschmidt et al (2012). Mutation is -1G to C, which changes the 3’ css to GCTACTCTGGAG/TT. Perhaps in addition to inactivating the 3’ss this mutation also increases the strength of the css, which might be why it was detected over the intron skip?

**Table 1 row 24. css -10 not predicted, plus a ss at -177 with 81 reads not seen**


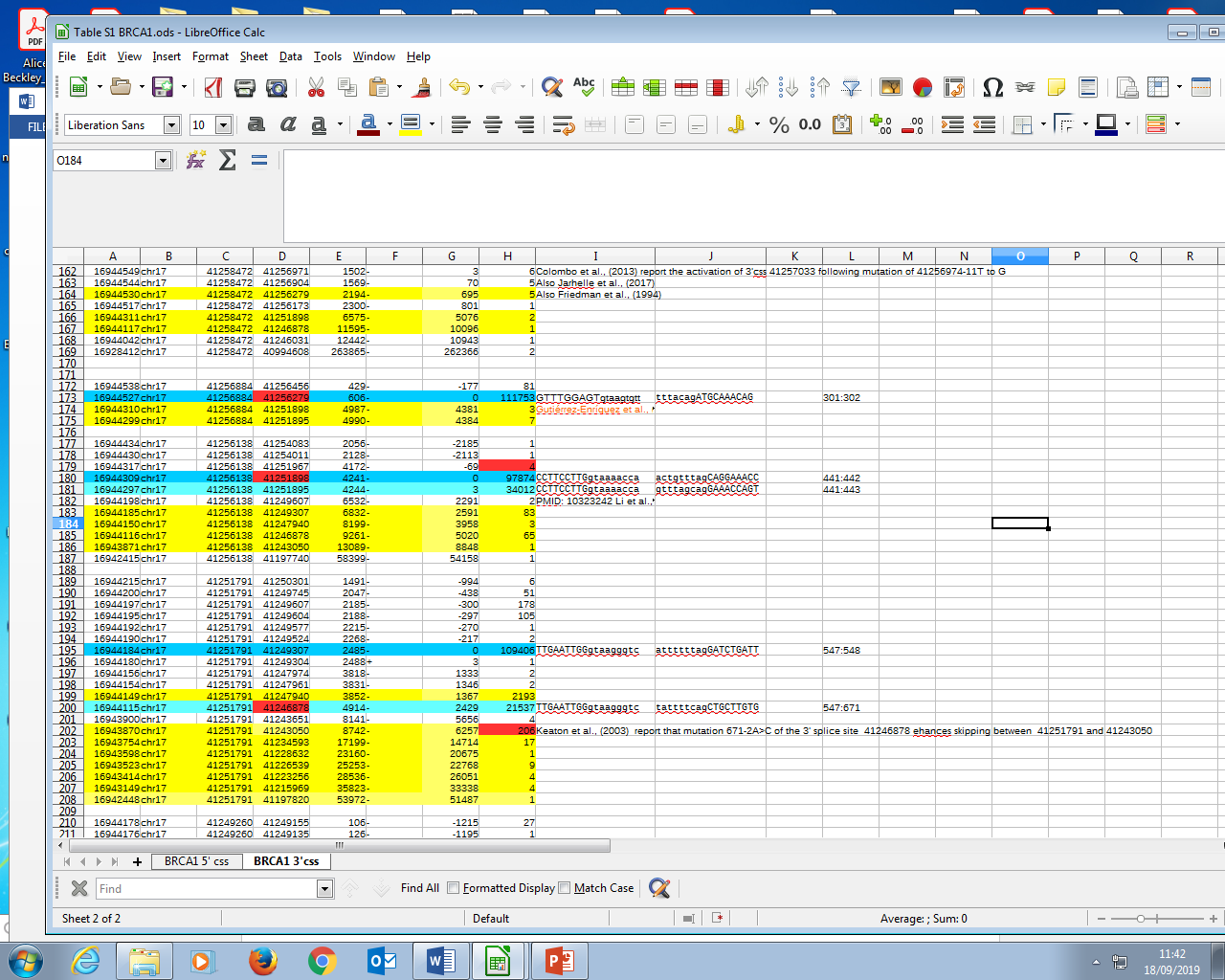


Gutiérrez-Enríquez et al., (2017) and Chen et al 2006 report the activation of a 3’css ten bases downstream of 41256279 following its mutation (ivs6 -1G>T and ivs6-2delA). Primers used were also capable of detecting a possible skip between 41256884 and 41251898.

Css they see is quite close to the mutation, which may account for its strong showing? Additional note: this css matches a bss in the larger SRAv2 database with 3 reads (below). The bss at -177 with 560 reads is a likely false positive.


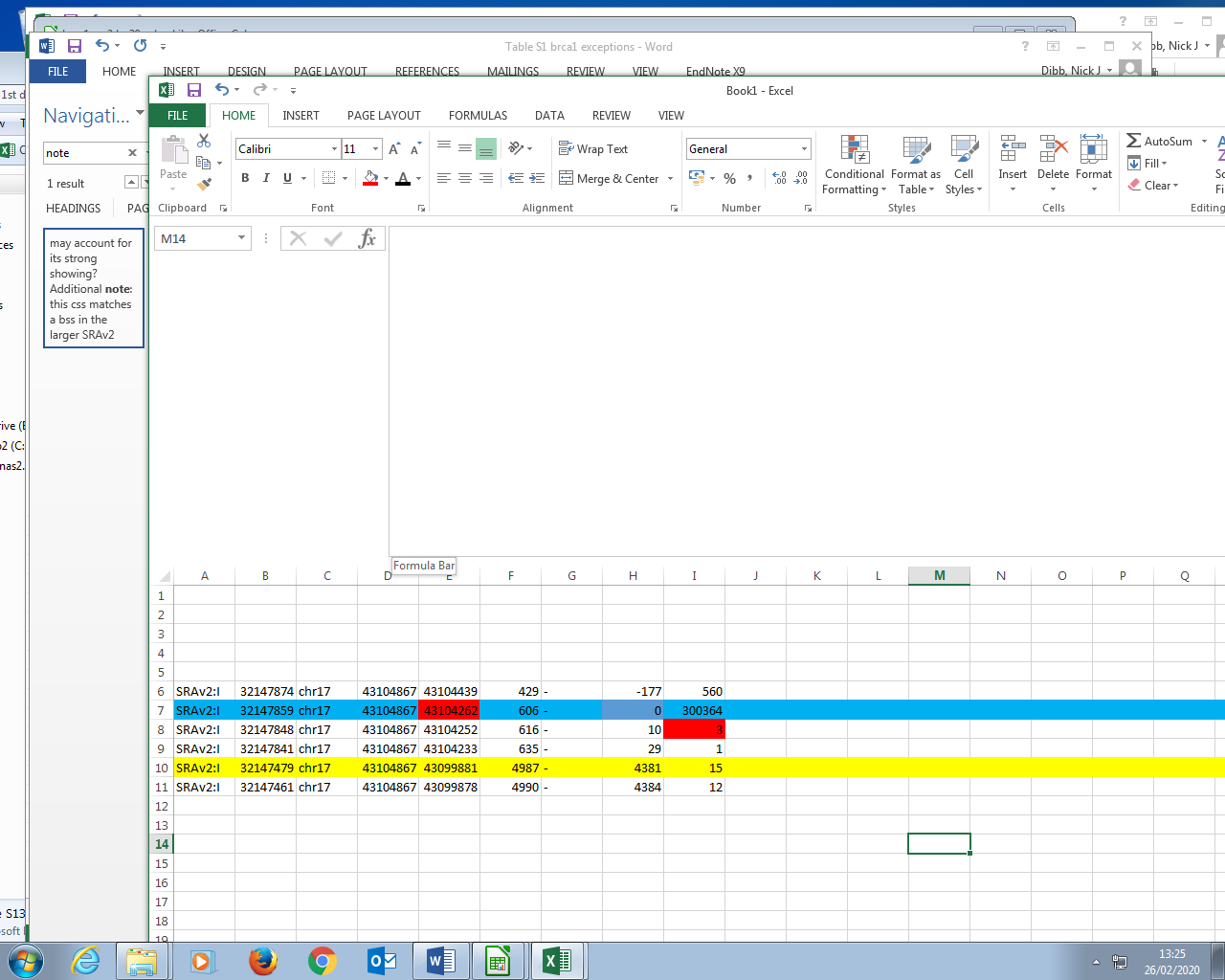


**Table 1 row 25. Single exon skip not detected despite having far more reads (83) than the reported css (4).**


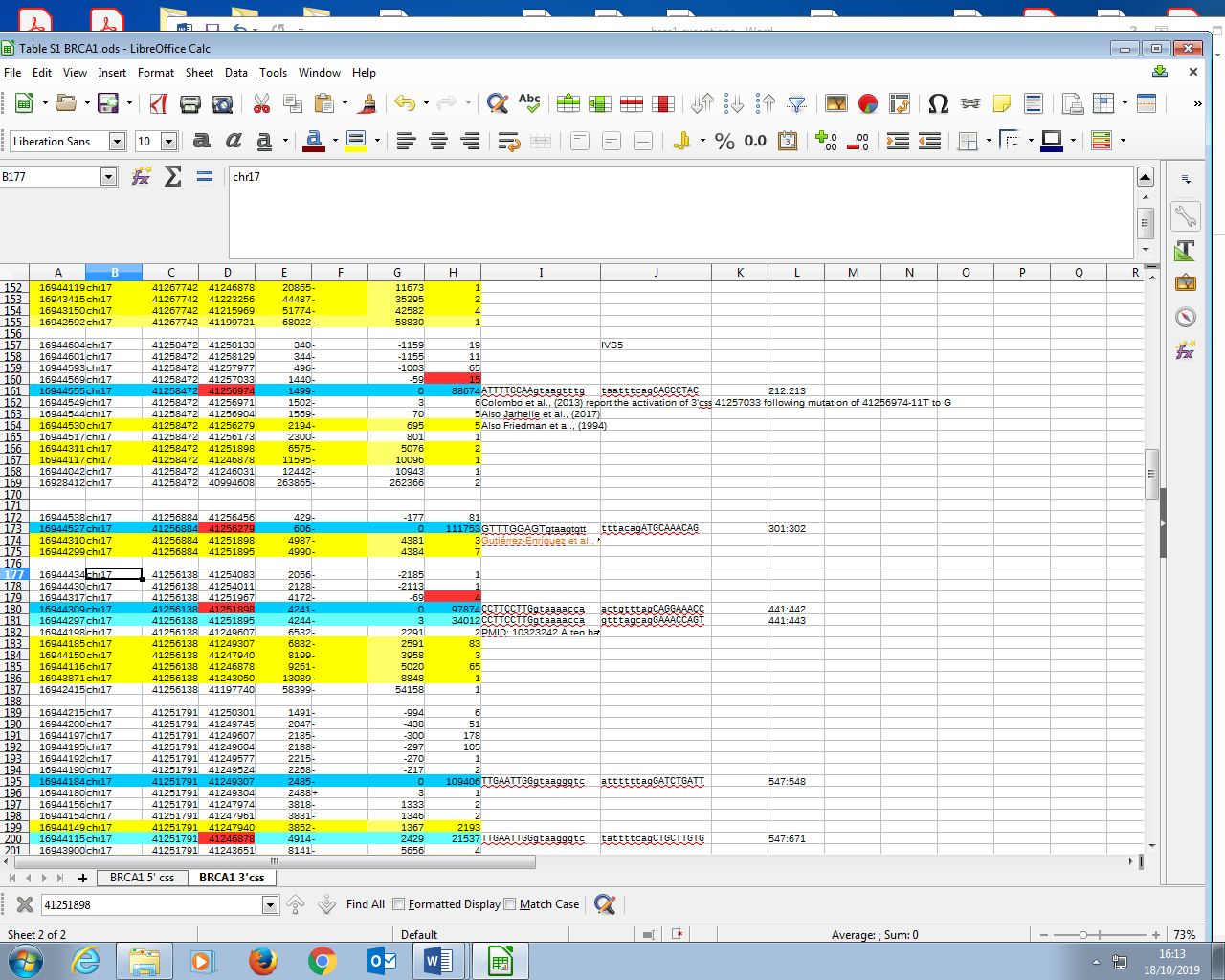


PMID: 10323242 A ten base deletion (41251922 to 41251912) activates the 3’css 41251967. The primers that were used for RT-PCR would not have detected a possible exon skip between 41256138 and 41249307.

**Table 1 row 31, possible false positive bss with 3 reads at 41209360 not detected**

Wappenschmidt et al (2012) PMID: 23239986 report strong exon skipping (41215349 to 4120315) and weaker activation of the 3’css 41209140 due to a mutation of the 3’ss 41209153-1 G to T.


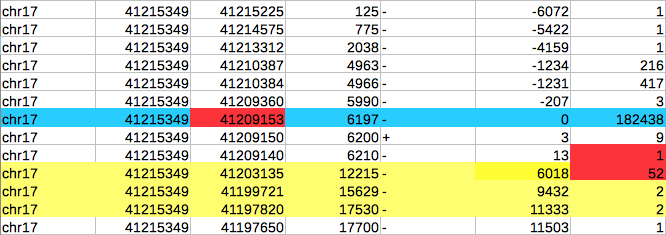


The strongest effect was exon skipping which agrees with the Snaptron reads. Bss 41209360 has more reads than bss 4129140 (which was activated as a weak css) but is further away from the mutated intron ss. Possible that the mutation may also have strengthened bss 4129140. Note: bss 41209150 is on the + transcript.

**Table 1 row 35, possible false positive bss with 26 reads not detected.** Baert et al., 2018 PMID: 29280214 report the activation of 3’css 41197809 following mutation of 3’ss 41197820 (-1G>A) The bss that matches the 3’css has 5 reads whereas another bss at -474 with 26 reads was not reported.


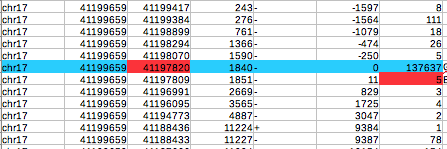


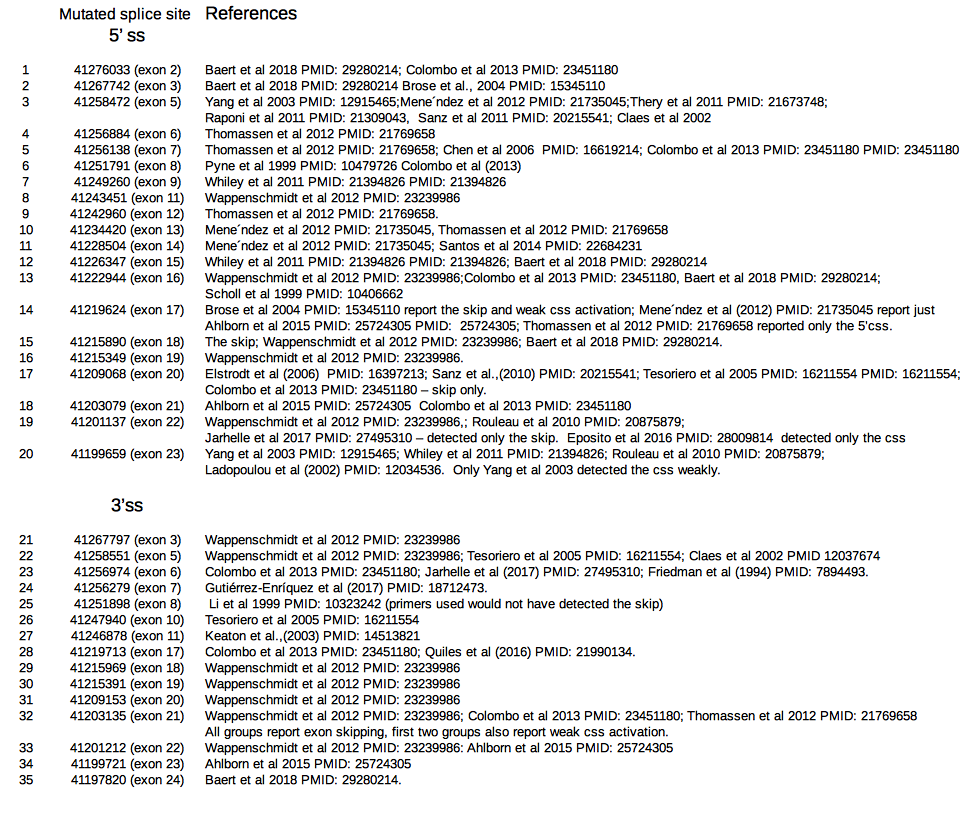

Supplement: Supplemental Material [file KRNB_A_2024031_SM7960.zip › Supplementary information/Table_S1_brca1_exceptions.docx]
